# Supplementary material for: Inter-individual and inter-site neural code conversion without shared stimuli
Source: Nat Comput Sci. 2025 Jul 11;5(7):534–46. doi: 10.1038/s43588-025-00826-5 (PMC12286860; doi:10.1038/s43588-025-00826-5)
Supplement: Supplementary file 1 — Supplementary Information [file 43588_2025_826_MOESM1_ESM.pdf]

nature computational science

Supplementary information https://doi.org/10.1038/s43588-025-00826-5

Inter-individual and inter-site neural code conversion without shared stimuli

In the format provided by the authors and unedited

Supplementary figures

Supplementary Figure 1 | Conversion accuracies of individual pairs.
Supplementary Figure 2 | Correlation in conversion accuracy between brain loss and content loss.
Supplementary Figure 3 | Comparison of conversion accuracies across functional alignment methods.
Supplementary Figure 4 | Converters trained using the loss from different DNN layers.
Supplementary Figure 5 | Comparison of conversion accuracies between whole VC and subarea-wise approaches.
Supplementary Figure 6 | Conversion performance between different visual subareas.
Supplementary Figure 7 | The effect of overlapping (shared) stimuli between converter and decoder trainings.
Supplementary Figure 8 | Auditory neural code conversion and inter-individual decoding.
Supplementary Figure 9 | Inter-individual decoding and image reconstruction using different functional alignment methods.
Supplementary Figure 10 | DNN feature decoding performance (pattern) with converters trained using the loss from different DNN layers.
Supplementary Figure 11 | DNN feature decoding performance (profile) with converters trained using the loss from different DNN layers.
Supplementary Figure 12 | The effect of stimulus overlap between converter and decoder trainings.
Supplementary Figure 13 | Reconstructed natural images of all pairs.
Supplementary Figure 14 | Reconstructed artificial images of all pairs.
Supplementary Figure 15 | Reconstructed natural images from different ROIs.
Supplementary Figure 16 | Reconstructed artificial images from different ROIs.
Supplementary Figure 17 | Evaluation of reconstructions based on feature correlations.
Supplementary Figure 18 | Converters consisting of different architectures.
Supplementary Figure 19 | Reconstruction performance with converters trained using the loss from different DNN layers.
Supplementary Figure 20 | Inter-site DNN feature decoding accuracies.
Supplementary Figure 21 | Inter-site image reconstructions for Deeprecon test data.
Supplementary Figure 22 | Inter-site image reconstructions for THINGS test data.
Supplementary Figure 23 | Inter-site image reconstructions for NSD test data.
Supplementary Figure 24 | DNN feature decoding performance using the CLIP-ViT decoder.
Supplementary Figure 25 | Reconstructed images with the CLIP-ViT decoder for all pairs.
Supplementary Figure 26 | The effect of training sample size for conversion.

Supplementary figures

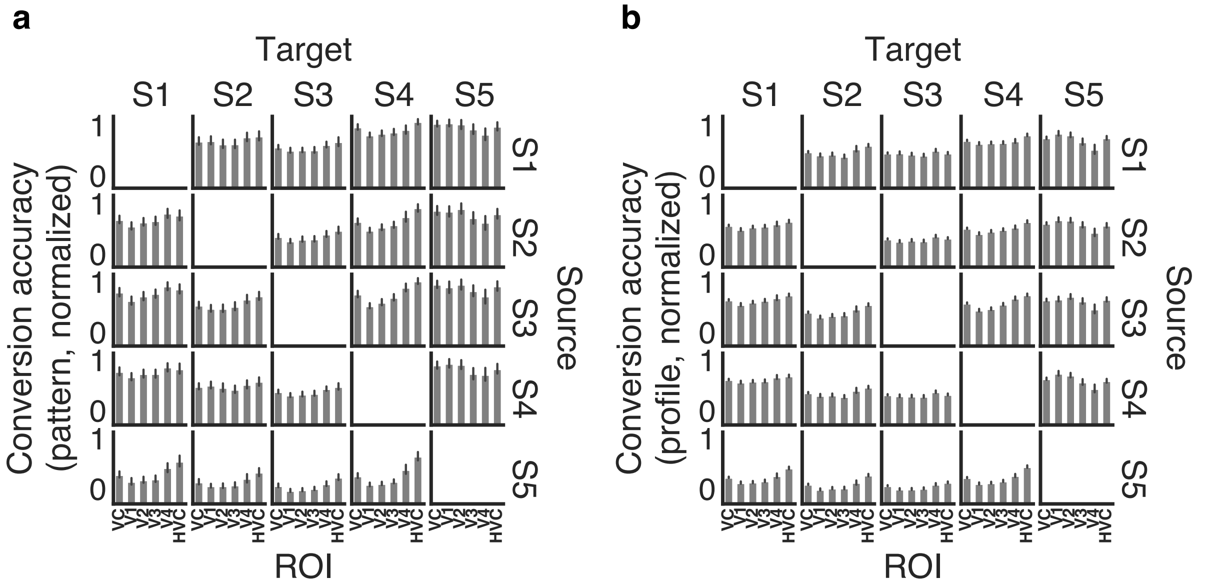

Supplementary Figure 1 | Conversion accuracies of individual pairs. **a**, Pattern correlation. The pattern correlation coefficients for 50 visual stimuli were used to calculate the mean conversion accuracy (pattern) and the 95% confidence interval (C.I.; represented by an error bar) in each pair of subjects. The mean conversion accuracies are shown for the whole visual cortex (VC) and its subareas, including V1, V2, V3, V4, and the higher visual cortex (HVC). **b**, Profile correlation. The profile correlation coefficients for individual voxels were used to calculate the mean conversion accuracy (profile) and the 95% confidence interval (error bar) in each pair of subjects. The mean conversion accuracies are shown for the VC and its subareas, including V1, V2, V3, V4, and HVC.

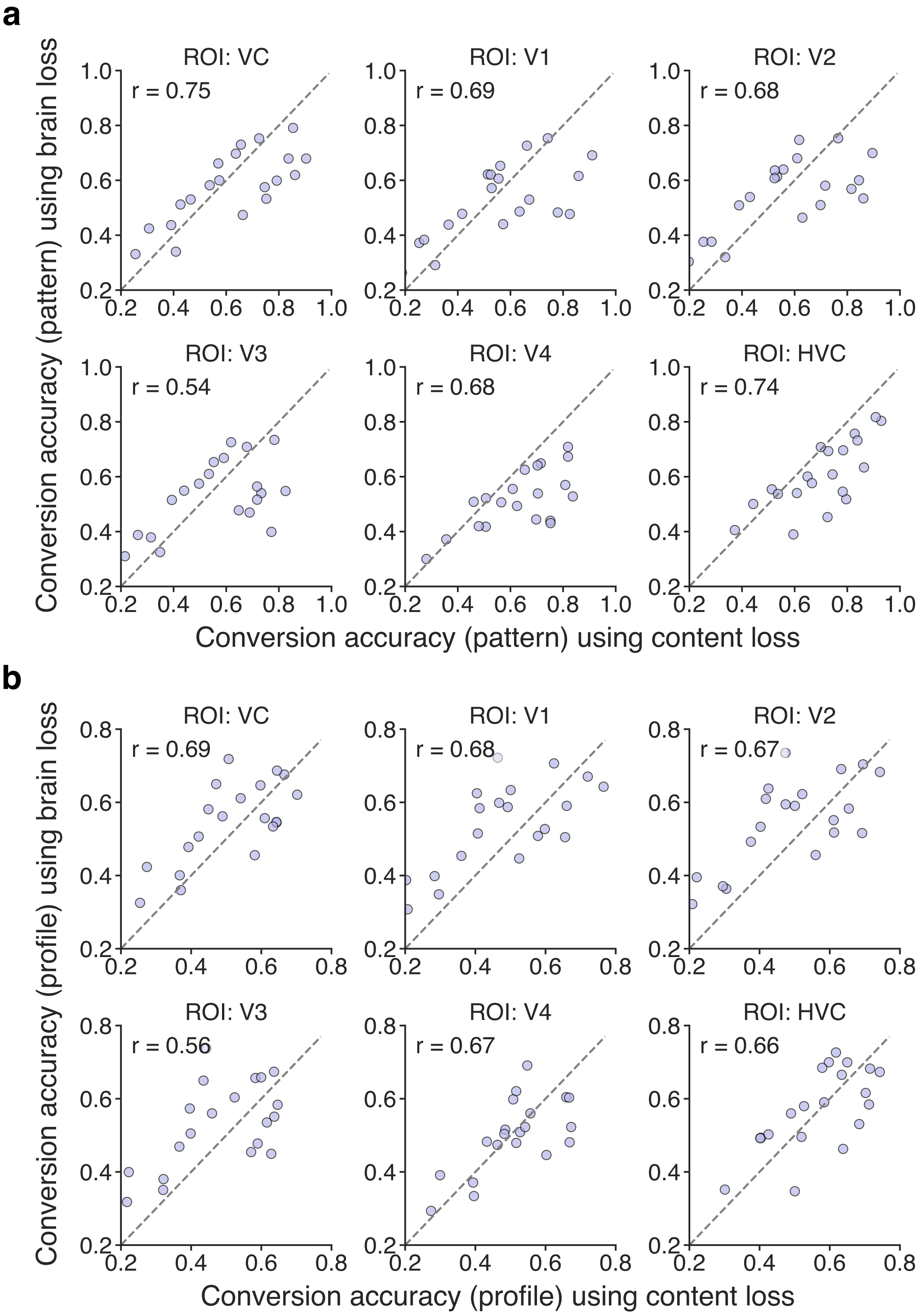

Supplementary Figure 2 | Correlation in conversion accuracy between brain loss and content loss. **a**, Scatter plots comparing conversion accuracies quantified using pattern correlations across different ROIs. The vertical axis shows accuracies obtained using brain loss, while the horizontal axis represents accuracies obtained using content loss. Each data point represents an individual subject pair, with a total of 20 pairs. The results are shown for the whole visual cortex (VC) and its subareas, including V1, V2, V3, V4, and the higher visual cortex (HVC). A diagonal reference line indicates where accuracies from both methods would be equal, allowing direct comparison of their performance. Pearson correlation coefficients (r) are displayed for each ROI to quantify the relationship between the two methods. **b**, Scatter plots comparing conversion accuracies quantified using profile correlations across different ROIs. The axes and reference lines follow the same conventions as in **a**, with correlation coefficients (r) shown for each ROI.

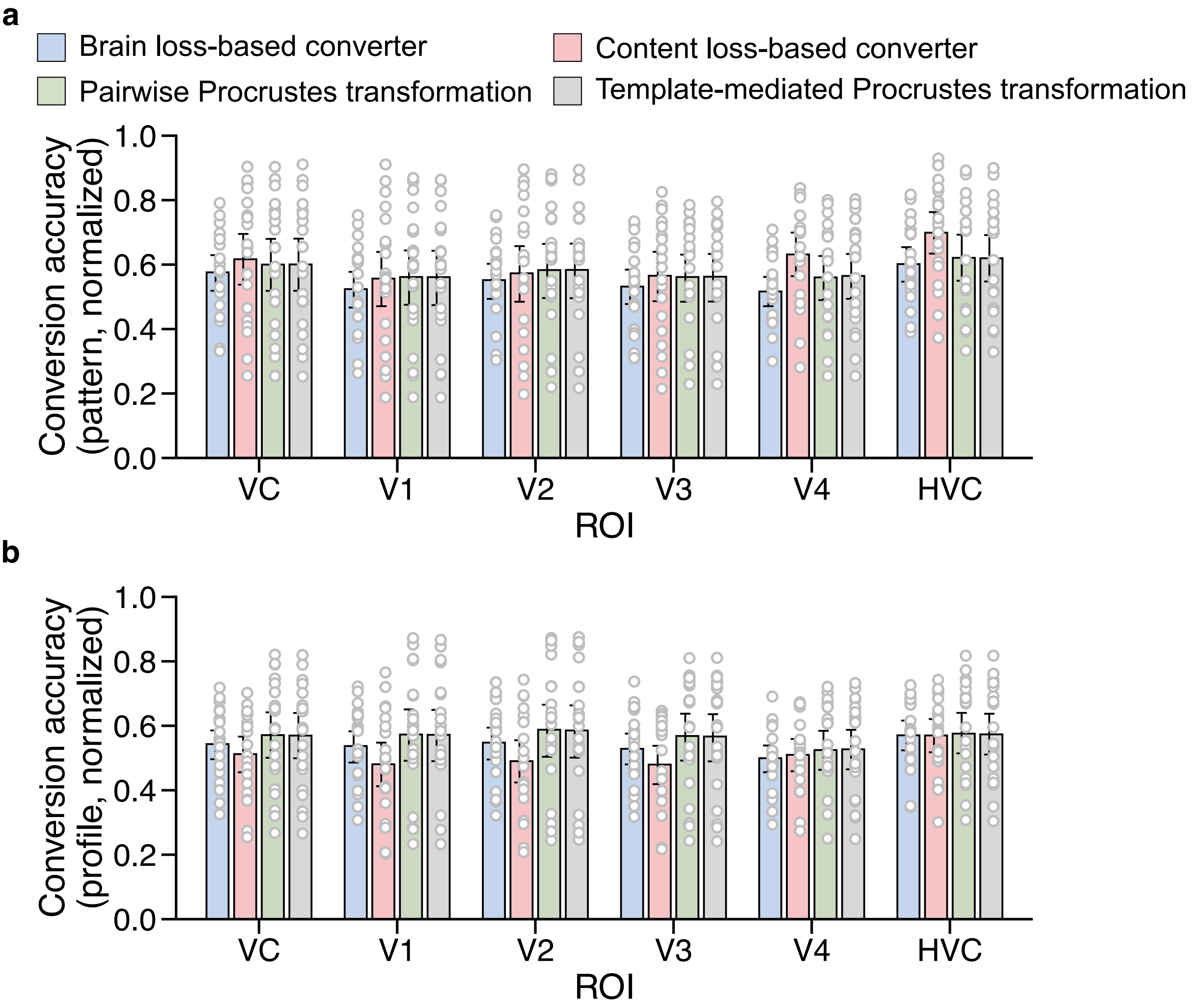

Supplementary Figure 3 | Comparison of conversion accuracies across functional alignment methods. **a**, Conversion accuracies quantified using pattern correlations for four different alignment methods in the VC and visual subareas. Correlation coefficients are shown for 20 subject pairs, where each dot represents a pair's mean correlation coefficient across stimuli. Bars indicate mean accuracies across all 20 pairs (error bars, 95% confidence intervals). The comparison includes the brain loss-based and content loss-based converters, along with two established methods: the pairwise Procrustes transformation and the template-mediated Procrustes transformation (hyperalignment[1]). The pairwise Procrustes transformation estimates an orthogonal matrix $\mathbf{M} \in \mathbb{R}^{n \times m}$ that minimizes the discrepancy between the source subject's brain activity pattern $\mathbf{x}_i \in \mathbb{R}^m$ and target subject's brain activity pattern $\mathbf{y}_i \in \mathbb{R}^n$, under the constraint $\mathbf{M}^\mathbf{T}\mathbf{M} = \mathbf{I}$, where $\mathbf{I}$ denotes the identity matrix. The orthogonal matrix $\mathbf{M}$ is optimized by minimizing the objective function $\mathcal{L}(\mathbf{M}) = \sum_i^N ||\mathbf{y}_i - (\mathbf{M}\mathbf{x}_i)||^2$, where N is the number of training samples. While the pairwise Procrustes transformation directly estimates the transformation matrix between a pair of subjects, the template-mediated Procrustes transformation first constructs a shared template across subjects through an iterative process of aligning individual data to a group-level representation. The final transformation for a pair of subjects is then achieved by sequentially mapping source activity to the template space and then to the target subject's brain space. The results show comparable conversion performance across the four alignment methods. **b**, Conversion accuracies quantified using profile correlations. The analysis follows the same methodology as in **a**, comparing all four alignment methods. Similar results were observed.

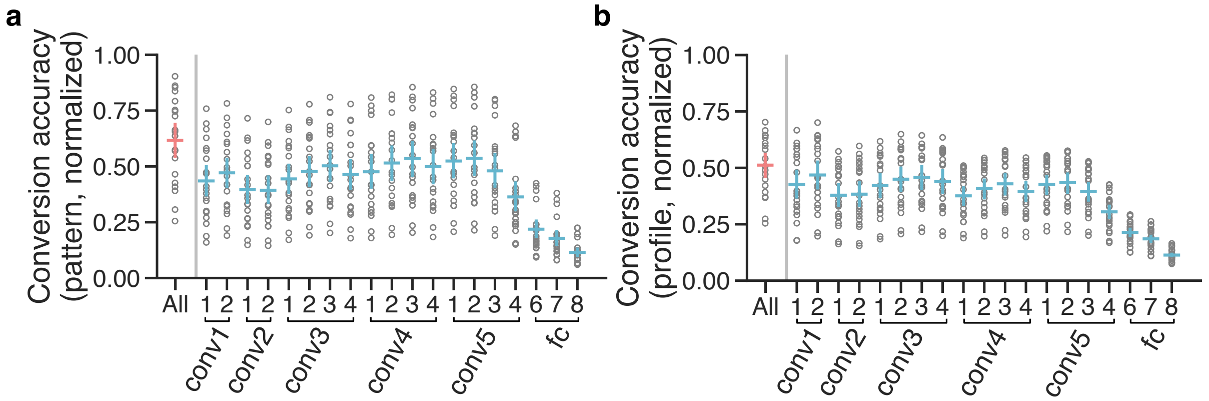

Supplementary Figure 4 | Converters trained using the loss from different DNN layers. **a**, Conversion accuracy measured by pattern correlation. To examine the significance of the use of multiple hierarchical DNN layers in converter training, we compared the conversion performance using the loss from different sets of DNN layers, including convolutional (conv) layers and fully connected (fc) layers of the VGG19 model. The pattern correlation coefficients calculated for 20 individual pairs (VC) are used to compare these converters trained using loss from different DNN layers. The horizontal bars represent the mean accuracies across the 20 pairs, with vertical bars representing the 95% confidence interval (C.I.), and each dot indicates an individual pair's mean correlation coefficient over stimuli. The results are shown with the converter trained using loss from all layers (All). It was observed that the loss from all layers resulted in the highest accuracy. The conversion accuracies were lower when using loss from the low-level and mid-level layers (conv1_1 to conv5_4). The high-level layers, specifically fc6, fc7, and fc8, showed the worst conversion accuracy. **b**, Conversion accuracy measured by profile correlation. The comparison is shown as in **a** but with profile correlation coefficients. Similar results were observed.

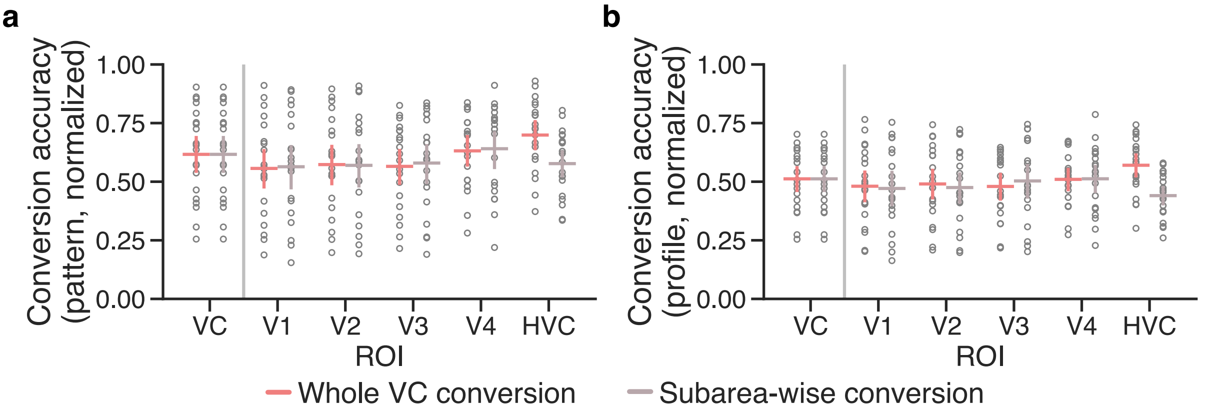

Supplementary Figure 5 | Comparison of conversion accuracies between whole VC and subarea-wise approaches. **a**, Conversion accuracies quantified using pattern correlations across different ROIs. The horizontal bars represent the mean accuracies across the 20 pairs, with vertical bars representing the 95% C.I., and each dot indicates an individual pair's mean correlation coefficient over stimuli. In the whole VC approach, the converter was trained to predict activity patterns in the target VC using the entire VC of the source subject, with accuracy evaluated separately for each ROI. In the subarea-wise approach, separate converters were trained for each ROI to predict activity patterns in target subareas using only the corresponding source subareas, with accuracy calculated for each ROI individually. This comparison evaluates the impact of incorporating prior anatomical information into the conversion process. Both methods achieve similar levels of performance, while in HVC, the subarea-wise method shows poorer performance. There were no significant differences in variance between the two methods across ROIs (Levene's test, $p > 0.05$ for all ROIs). The results indicate that incorporating anatomical constraints provides no substantial benefit and suggest that the flexible mapping afforded by the whole VC method may be advantageous. **b**, Conversion accuracies quantified using profile correlations across different ROIs. The analysis follows the same methodology as in **a**, comparing whole VC and subarea-wise approaches. Similar results to **a** were observed.

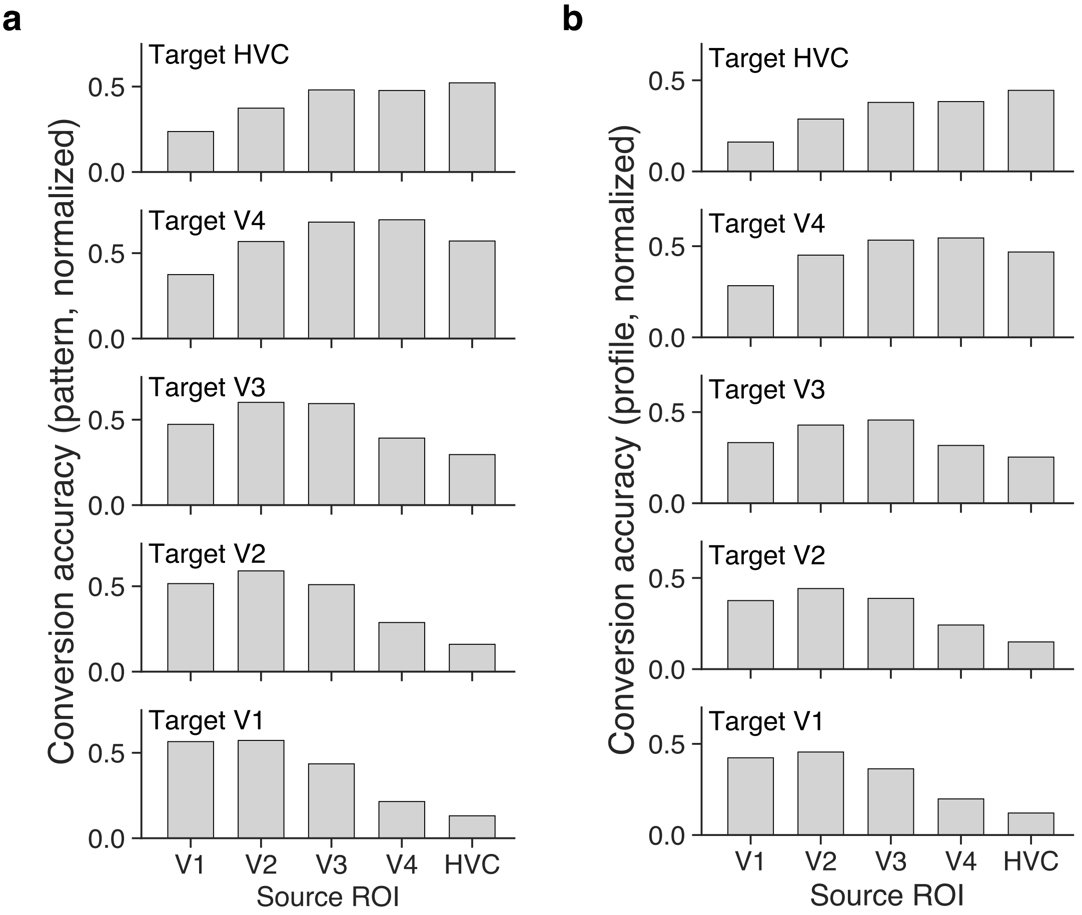

Supplementary Figure 6 | Conversion performance between different visual subareas. a, Conversion accuracies quantified using pattern correlations for all combinations of subareas between source and target subjects. Unlike the subarea-wise conversion within corresponding ROIs shown in Supplementary Figure 5, this analysis involved predicting activity patterns in a target subarea using different source subareas (e.g., using source V1, V2, V3, V4, or HVC individually to predict target V1). The results from a representative subject are shown. In most subareas, the highest conversion accuracy is achieved using the corresponding source subarea, suggesting that neural code conversion accuracy can potentially serve as a quantitative measure of homology between brain areas. **b**, Conversion accuracies quantified using profile correlations. The analysis follows the same methodology as in **a**, yielding similar results.

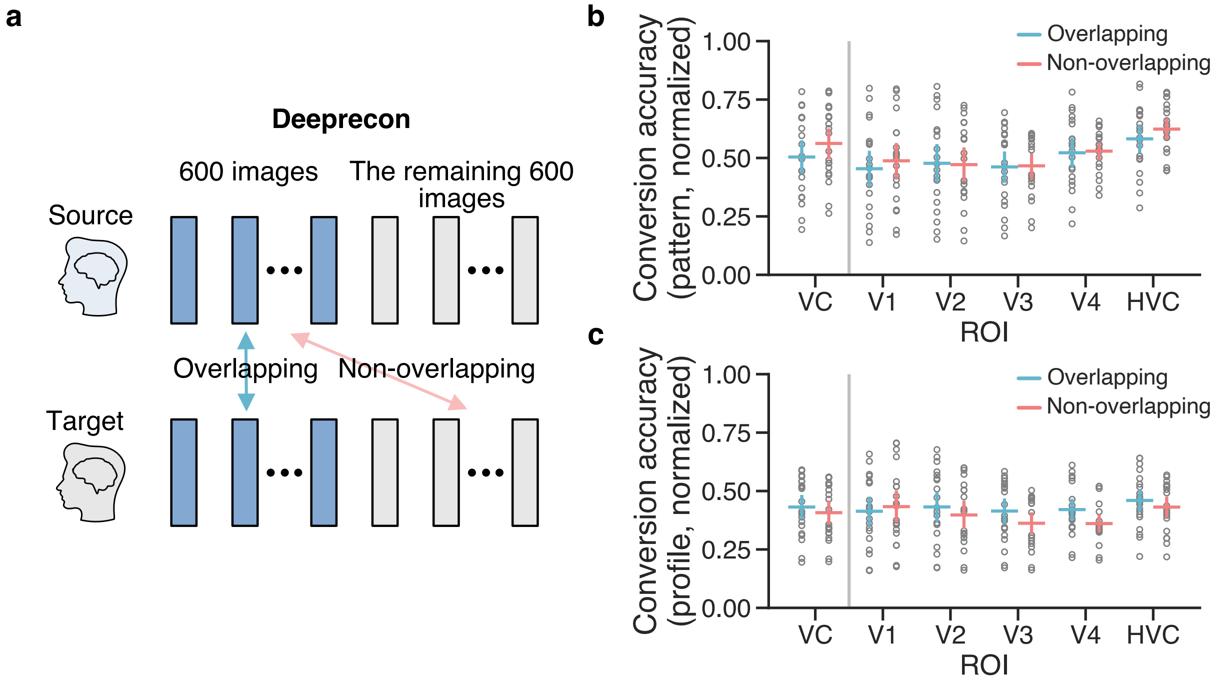

Supplementary Figure 7 | The effect of overlapping (shared) stimuli between converter and decoder trainings. **a**, Non-overlapping stimuli for source and target subjects. To examine whether the content loss approach remains effective without shared stimuli, we performed neural code conversion with no stimulus overlap between the converter and decoder trainings. We randomly divided the training samples from the Deeprecon dataset into two distinct halves based on the categories of stimuli. The source subject was provided with 3000 training samples (600 images from 75 randomly selected categories out of 150 categories, with five repetitions of each image), and the target subject was given a different set of 3000 training samples (the remaining 600 images with five repetitions each), a condition we refer to as "Non-overlapping". This strategy was designed to prevent any pairing in their brain activity patterns. For comparison, we also performed neural code conversion under an "Overlapping" condition, where the target subject used training samples the same as the source subject, resulting in overlapping stimuli between the converter and decoder trainings. **b**, Conversion accuracy measured by pattern correlation. Distributions of the pattern coefficients of 20 pairs are shown for the VC and visual subareas. The horizontal bars represent the mean accuracies across the 20 pairs, with vertical bars representing the 95% C.I., and each dot indicates an individual pair's mean correlation coefficient over stimuli. The content loss-based converter demonstrates comparable performance under both conditions across ROIs. There were no significant differences in variance between the two conditions in most ROIs (Levene's test, pattern correlation: $p > 0.05$ for all ROIs except V4, where $p = 0.035$). **c**, Conversion accuracy measured by profile correlation. The two conditions are compared as in **b** but with profile correlation coefficients. Similar results to **b** were observed. There were no significant differences in variance between the two conditions across ROIs (Levene's test, $p > 0.05$ for all ROIs).

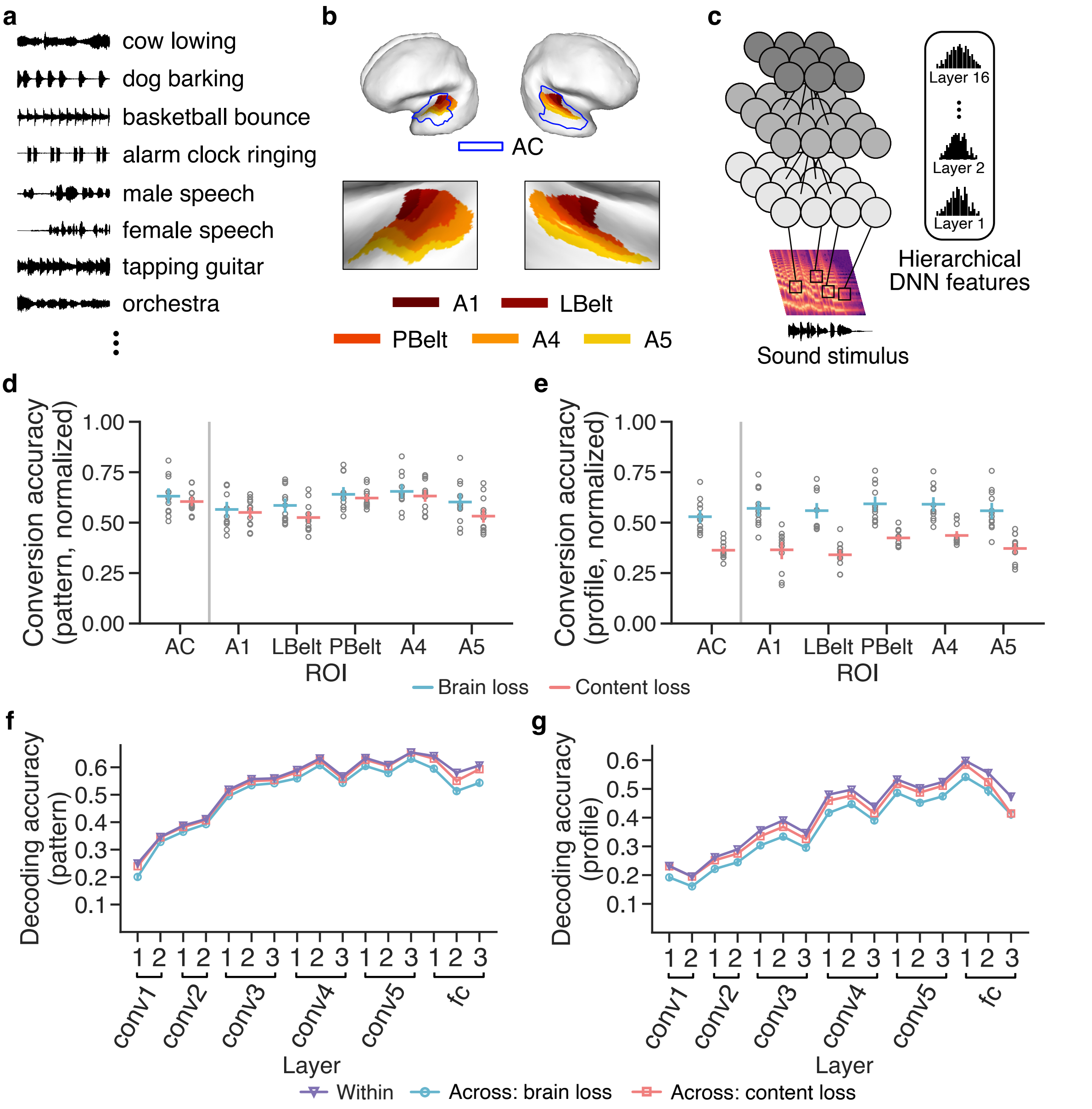

Supplementary Figure 8 | Auditory neural code conversion and inter-individual decoding. **a**, Sound stimuli from the DeepSoundRecon dataset. The DeepSoundRecon dataset[2] consists of 1,200 training audio clips and 50 test audio clips (8 s each) with corresponding fMRI responses (four repetitions for each training clip and eight repetitions for each test clip) from five subjects. Subject 1 (S1), a pilot subject, was excluded due to the exploratory nature of their data[2]. **b**, The ROIs of the auditory cortex. Whole-brain fMRI data were collected while subjects listened to auditory stimuli. The analysis used ROIs defined by the dataset, including the whole auditory cortex (AC), A1, LBelt, and PBelt from the early auditory cortex, as well as A4 and A5 from the auditory association cortex. **c**, Hierarchical DNN features as auditory contents. DNN features were extracted from sound spectrograms using a VGGish-ish DNN model with 13 convolutional (conv) layers and 3 fully connected (fc) layers[3]. The DNN feature decoders and the converters (brain loss- and content loss-based) were trained and evaluated following the same procedures used for the vision tasks. **d**, Conversion accuracies measured by pattern correlation. Distributions of the pattern correlation coefficients of 12 individual pairs are shown for the AC and auditory subareas. The horizontal bars represent the mean accuracies across the 12 pairs, with vertical bars representing the 95% C.I., and each dot indicates an individual pair's mean correlation coefficient over stimuli. The content loss-based converter achieved comparable performance to the brain loss-based converter. **e**, Conversion accuracies measured by profile correlation. The analysis follows the same procedure as in **d**, with results showing that the content loss-based method achieves generally lower performance than the brain loss-based approach. **f**, Feature decoding accuracy measured by pattern correlation. The mean pattern correlation for each layer of the VGGish-ish model is shown for the Within, Brain loss, and Content loss conditions (AC; error bars, 95% C.I. from four subjects for the Within condition, and from 12 pairs for the Brain loss and Content loss conditions). The content loss-based converter achieves comparable decoding performance to both the brain loss-based approach and the within-individual approach. **g**, Feature decoding accuracy measured by profile correlation. The three conditions are compared as in **f** but with profile correlation coefficients. The analysis follows the same procedure as in **f**, yielding similar results.

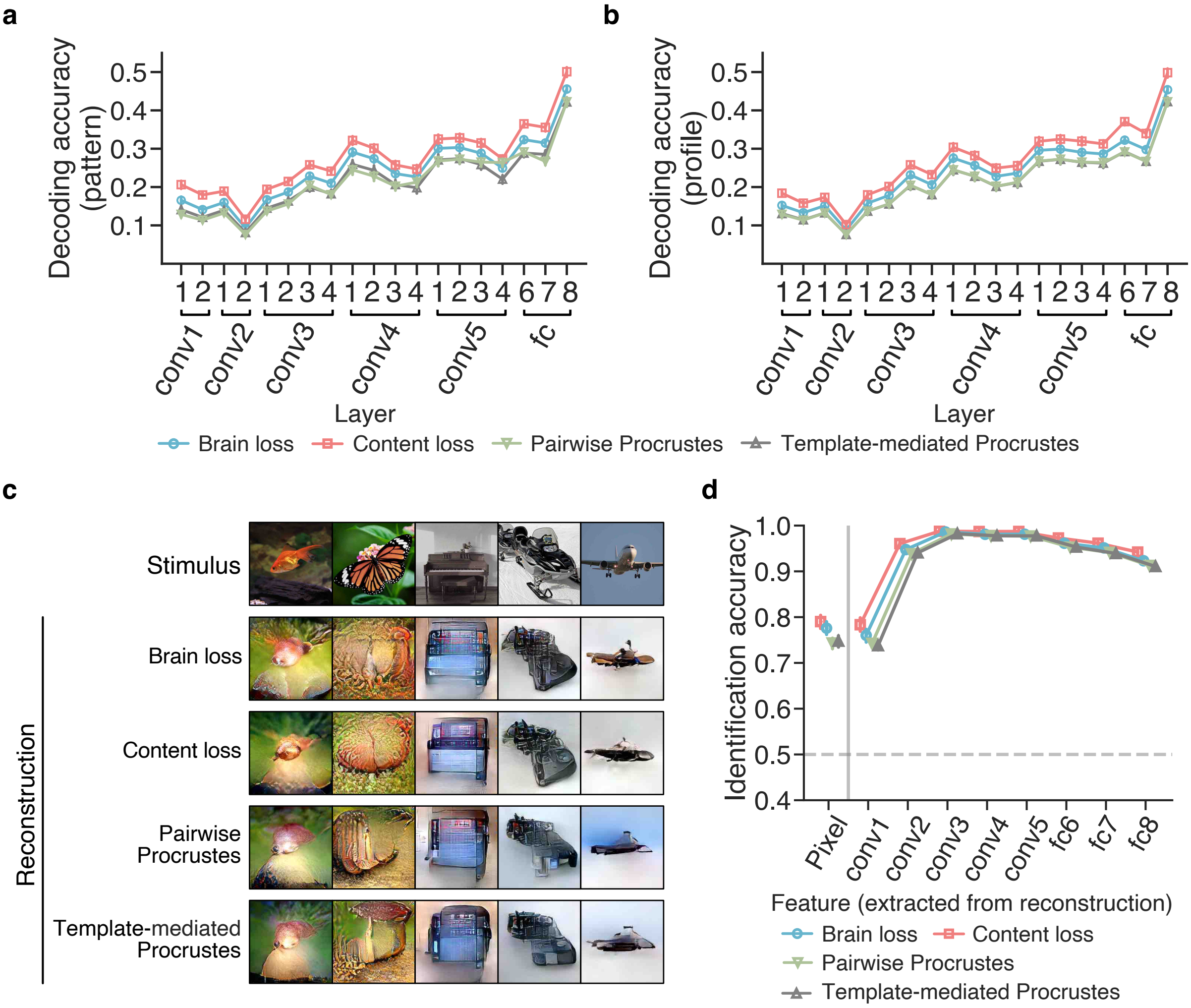

Supplementary Figure 9 | Inter-individual decoding and image reconstruction using different functional alignment methods. **a**, Feature decoding accuracy measured by pattern correlation. The DNN features from the convolutional (conv) layers and fully connected (fc) layers of the VGG19 model are analyzed. The mean pattern correlation for each layer is shown for the brain loss-based converter, content loss-based converter, pairwise Procrustes transformation, and template-mediated Procrustes transformation (VC; error bars, 95% C.I. from 20 pairs for each condition). **b**, Feature decoding accuracy measured by profile correlation. The four conditions are compared as in **a** but with profile correlation coefficients. **c**, Reconstructions of natural images. The reconstructions under the four analytical conditions for each stimulus image were all from the same source subject (VC; source: Subject 1, target: Subject 2). For copyright reasons, some stimulus images have been replaced with visually similar alternatives. **d**, Identification accuracy of natural images. Pairwise identification was performed using the pixel values and the extracted DNN feature values from the reconstructions. The DNN features were extracted from eight layers of the AlexNet model, including convolutional (conv) layers and fully connected (fc) layers. The mean identification accuracy was calculated over all reconstructed images for each individual pair, and then averaged across all pairs to obtain the group-level mean (error bars, 95% C.I. from 20 pairs; dashed lines, 50% chance level).

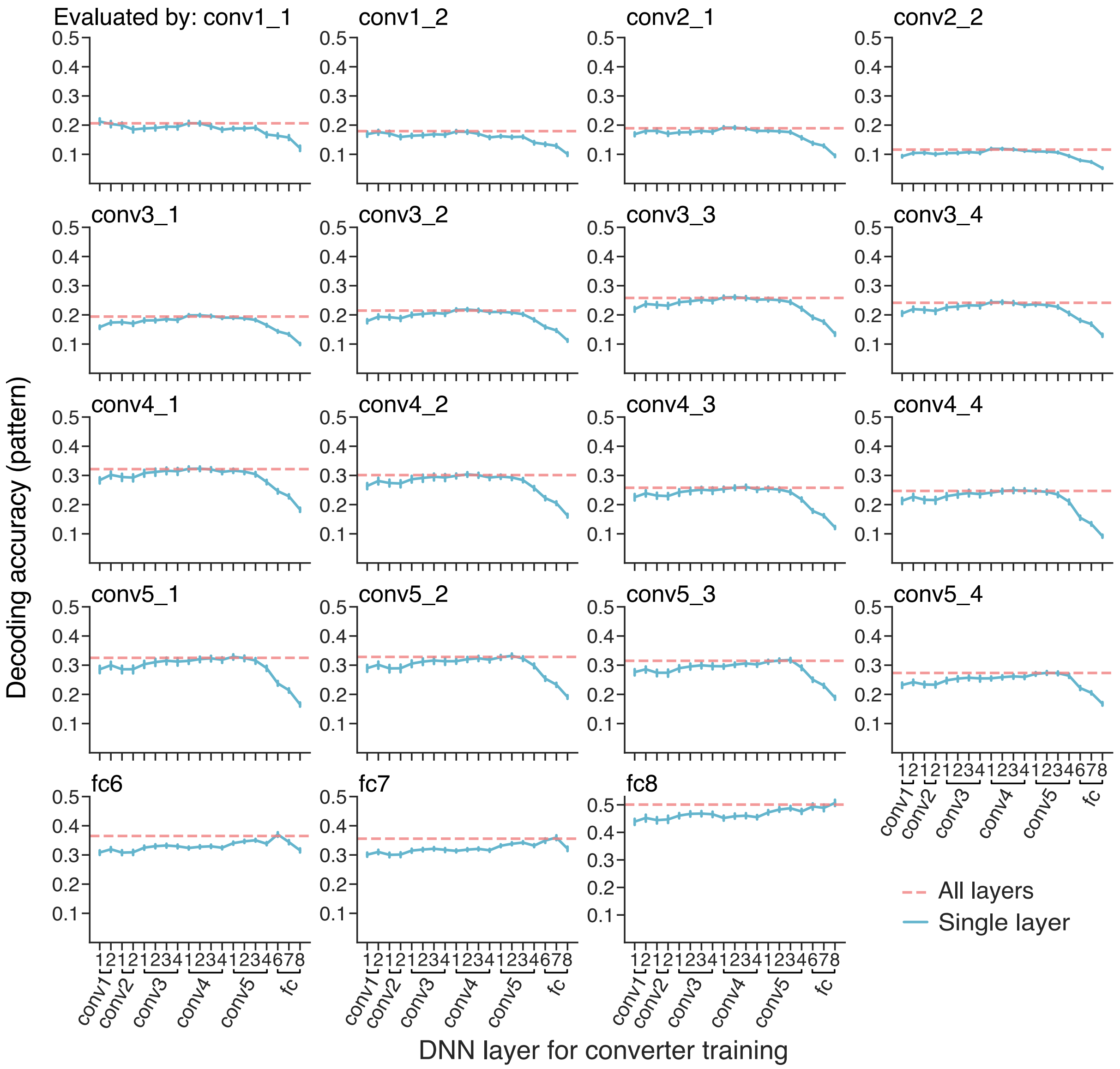

Supplementary Figure 10 | DNN feature decoding performance (pattern) with converters trained using the loss from different DNN layers. Converters were trained using the loss from different layers of the VGG19 model. The “All layers” refers to converters trained using the loss from all DNN layers, while the “single layer” corresponds to converters trained using the loss from single DNN layers. The feature decoding performance of the converted brain activity was evaluated by the mean pattern correlation coefficient at each layer of the VGG19 model (VC; error bars, 95% C.I. from 20 pairs).

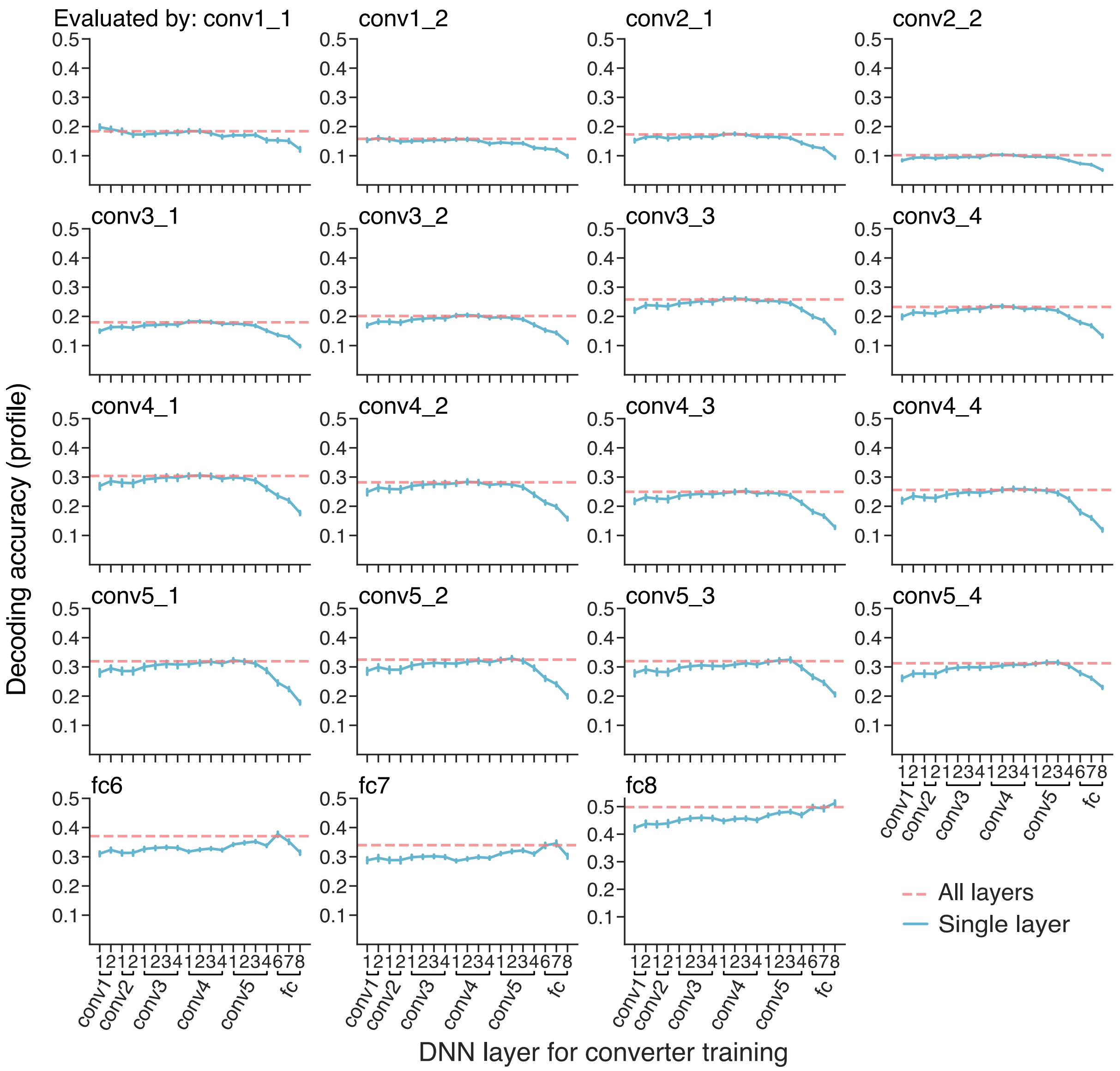

Supplementary Figure 11 | DNN feature decoding performance (profile) with converters trained using the loss from different DNN layers. The decoded features, obtained from the converters and decoders described in Supplementary Figure 10, were evaluated using profile correlation coefficients. The feature decoding performance was evaluated by the mean profile correlation coefficient at each layer of the VGG19 model (VC; error bars, 95% C.I. from 20 pairs).

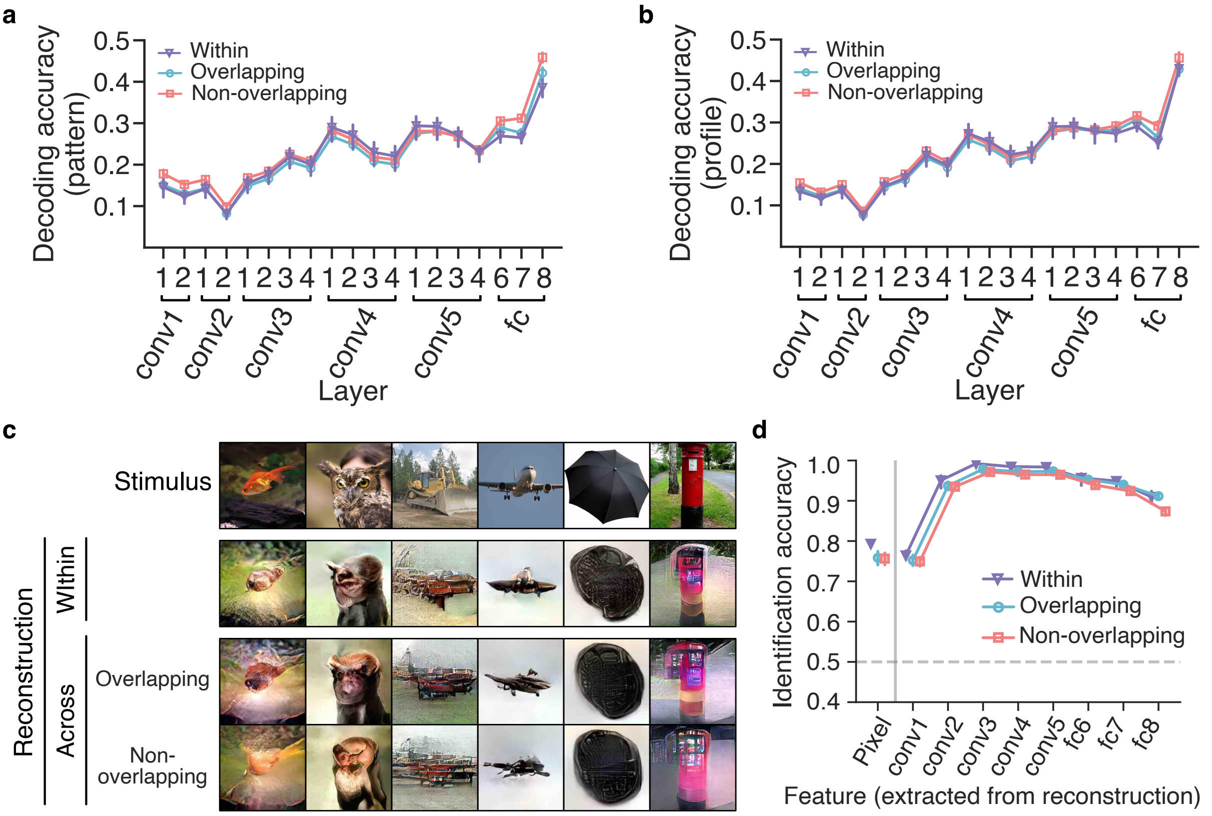

Supplementary Figure 12 | The effect of stimulus overlap between converter and decoder trainings. **a**, Feature decoding accuracy measured by pattern correlation. The mean pattern correlation for each layer of the VGG19 model is shown for the Within, Overlapping, and Non-overlapping conditions (VC; error bars, 95% C.I. from five subjects for the Within condition, and from 20 pairs for the Overlapping, and Non-overlapping conditions). **b**, Feature decoding accuracy measured by profile correlation. The three conditions are compared as in **a** but with profile correlation coefficients. **c**, Reconstructions. The reconstructions under three analytical conditions for each stimulus were all from the same source subject (VC; source: Subject 1, target: Subject 2). For copyright reasons, some stimulus images have been replaced with visually similar alternatives. **d**, Identification accuracy. Pairwise identification was performed using the pixel values and the extracted DNN feature values from the reconstructions. The mean identification accuracy was calculated over all reconstructed images for each subject or individual pair, and then averaged across all subjects or pairs to obtain the group-level mean (error bars, 95% C.I. from 20 pairs; dashed lines, 50% chance level).

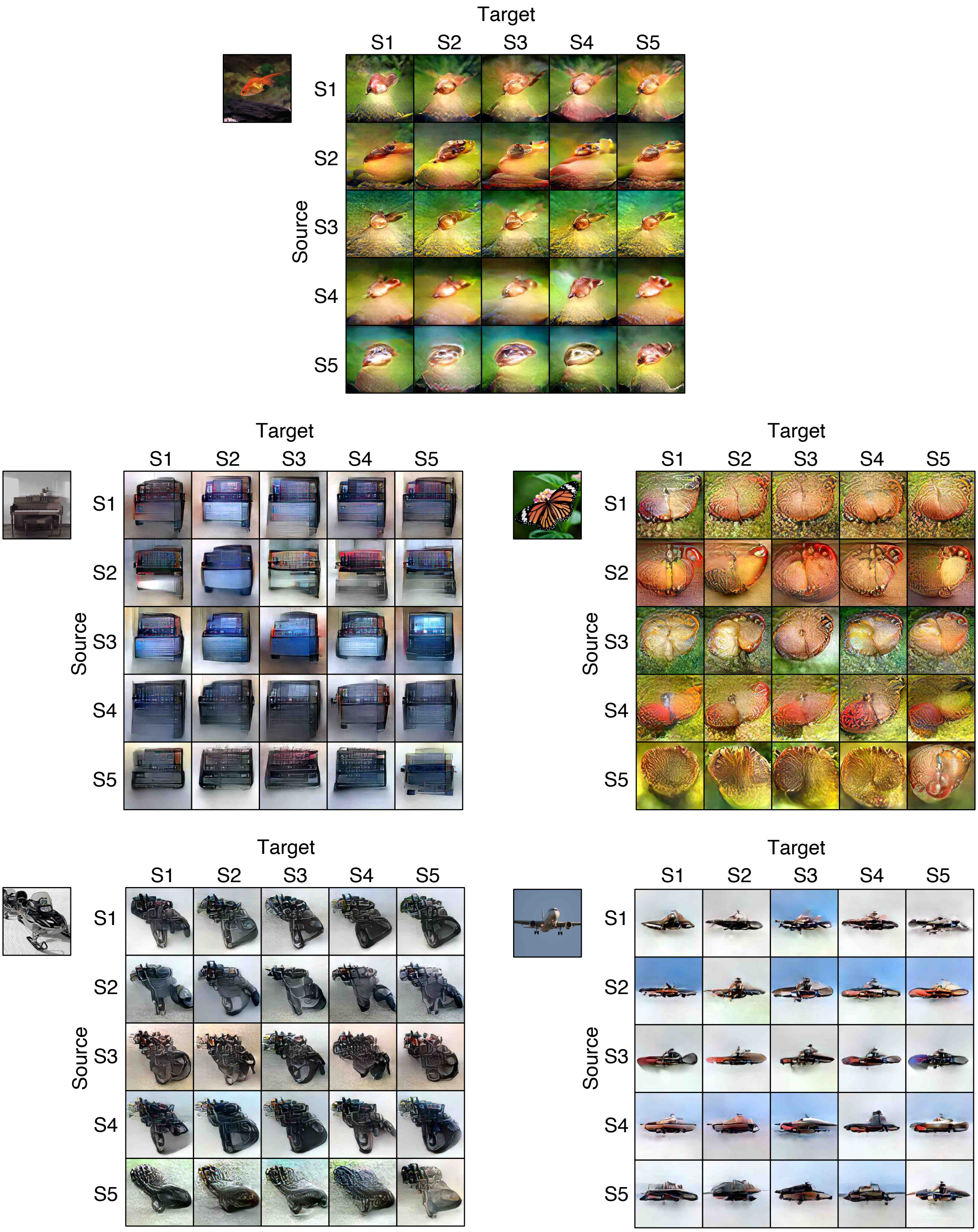

Supplementary Figure 13 | Reconstructed natural images of all pairs. The reconstructions are shown for each pair of subjects for each stimulus image. The diagonal images in each block are reconstructed in the Within condition. All reconstructions were generated using the whole visual cortex (VC). For copyright reasons, some stimulus images have been replaced with visually similar alternatives.

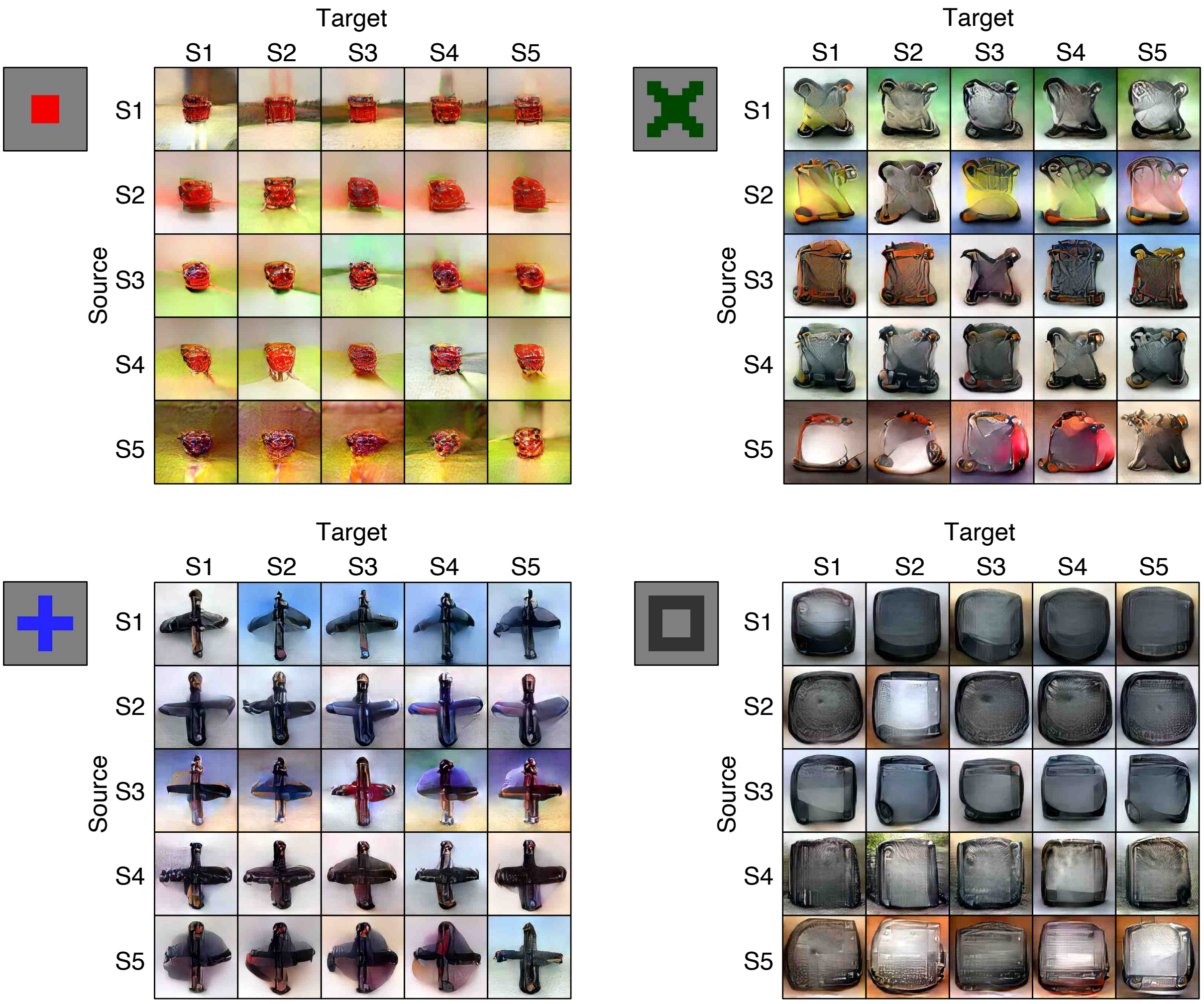

Supplementary Figure 14 | Reconstructed artificial images of all pairs. The reconstructions are shown for each pair of subjects for each stimulus image. The diagonal images in each block are reconstructed in the Within condition. All reconstructions were generated using the whole visual cortex (VC).

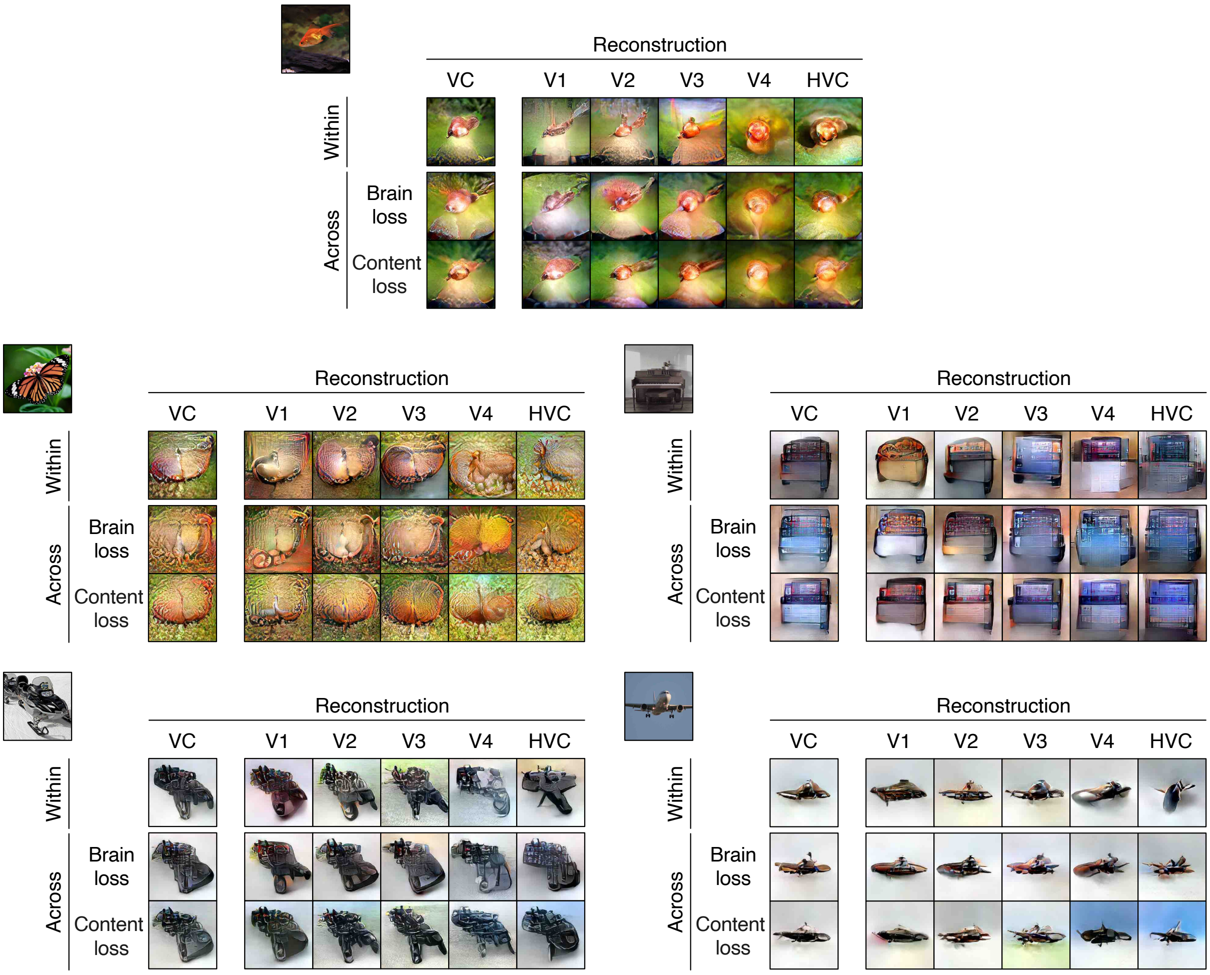

Supplementary Figure 15 | Reconstructed natural images from different ROIs. Reconstructions were generated from different ROIs in the Within, Brain loss, and Content loss conditions. All reconstructions are from the same source subject (source: Subject 1, target: Subject 2). Compared to reconstructions from the VC, those from other ROIs show a slight degradation in quality but still reflect the main characteristics of the presented images. For copyright reasons, some stimulus images have been replaced with visually similar alternatives.

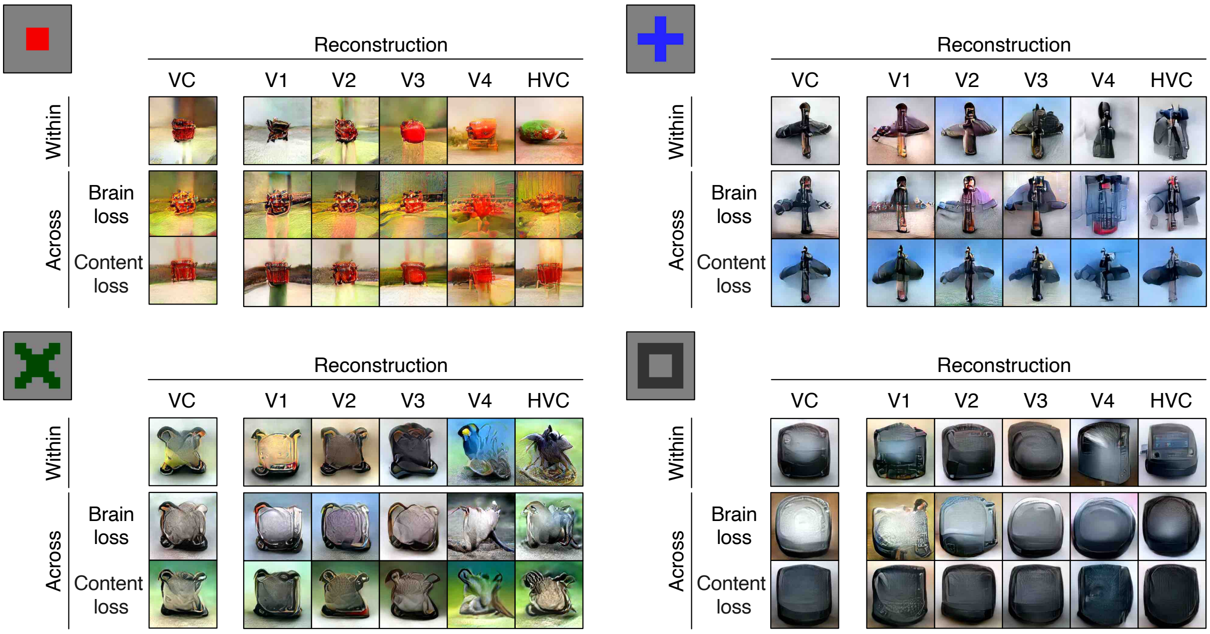

Supplementary Figure 16 | Reconstructed artificial images from different ROIs. Reconstructions were generated from different ROIs in the Within, Brain loss, and Content loss conditions. All reconstructions are from the same source subject (source: Subject 1, target: Subject 2). Similar results to Supplementary Figure 15 were observed.

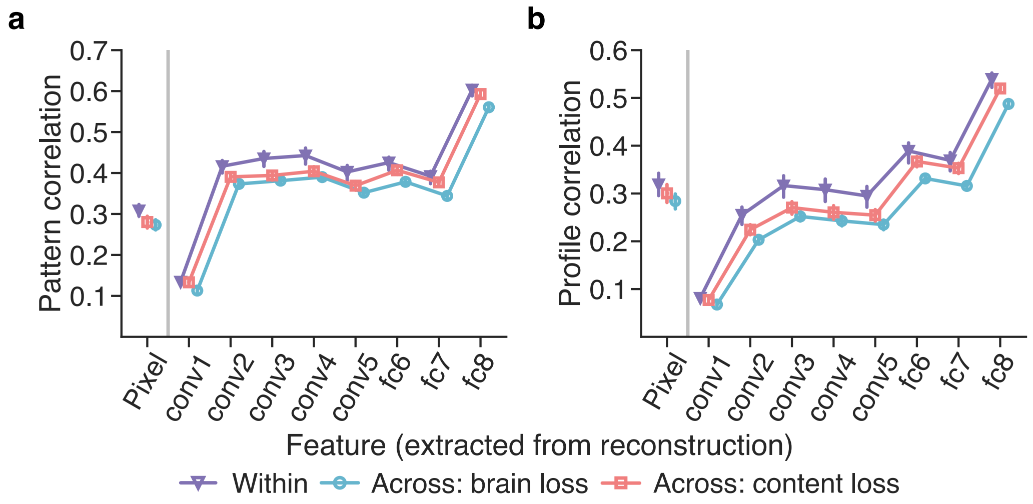

Supplementary Figure 17 | Evaluation of reconstructions based on feature correlations.
a, Pattern correlation of natural images. Pattern correlation between the reconstruction and the presented image was calculated using the pixel values and the extracted DNN feature values (AlexNet) from the reconstructions. The mean pattern accuracy was calculated across all subjects or individual pairs (error bars, 95% C.I. from five subjects or 20 pairs; dashed lines, 50% chance level). The content loss-based converter consistently outperformed the brain loss-based converter across all layers **b**, Profile correlation of natural images. The three conditions are compared as in **a** but with profile correlation, yielding similar results.

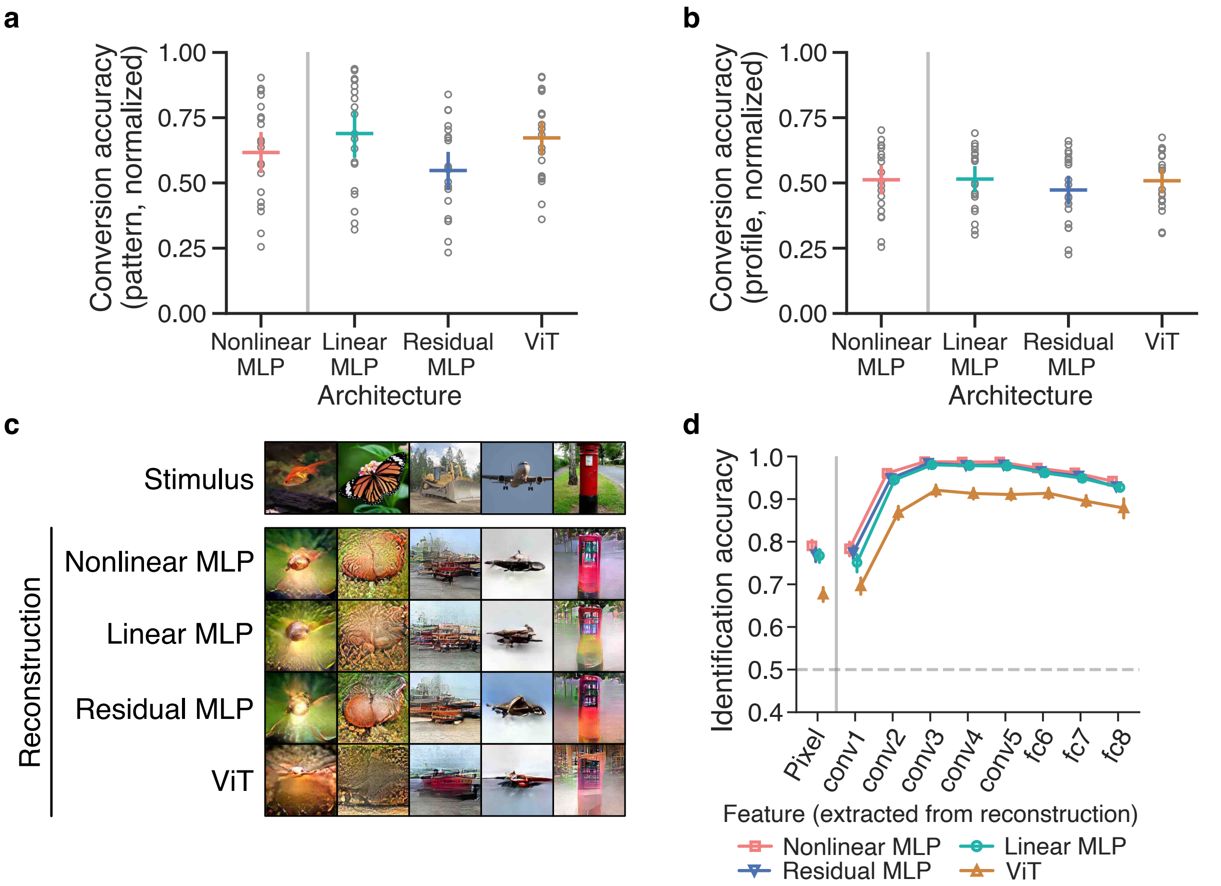

Supplementary Figure 18 | Converters consisting of different architectures. **a**, Conversion accuracies measured by pattern correlation. Nonlinear MLP, Linear MLP, Residual MLP, and Visual Transformer (ViT) converters are compared using the profile correlation coefficients calculated for 20 individual pairs (VC). The horizontal bars represent the mean accuracies across the 20 pairs, with vertical bars representing the 95% C.I., and each dot indicates an individual pair's mean correlation coefficient over stimuli. **b**, Conversion accuracies measured by profile correlation. The four architectures are compared as in **a** but with profile correlation coefficients. **c**, Reconstructions. Examples for source Subject 1 and target Subject 2 (VC) are shown. For copyright reasons, some stimulus images have been replaced with visually similar alternatives. **d**, Identification accuracy. The four architectures are compared using the identification accuracies based on pixel and extracted DNN feature values (error bars, 95% C.I. from 20 pairs; dashed lines, chance level = 50%).

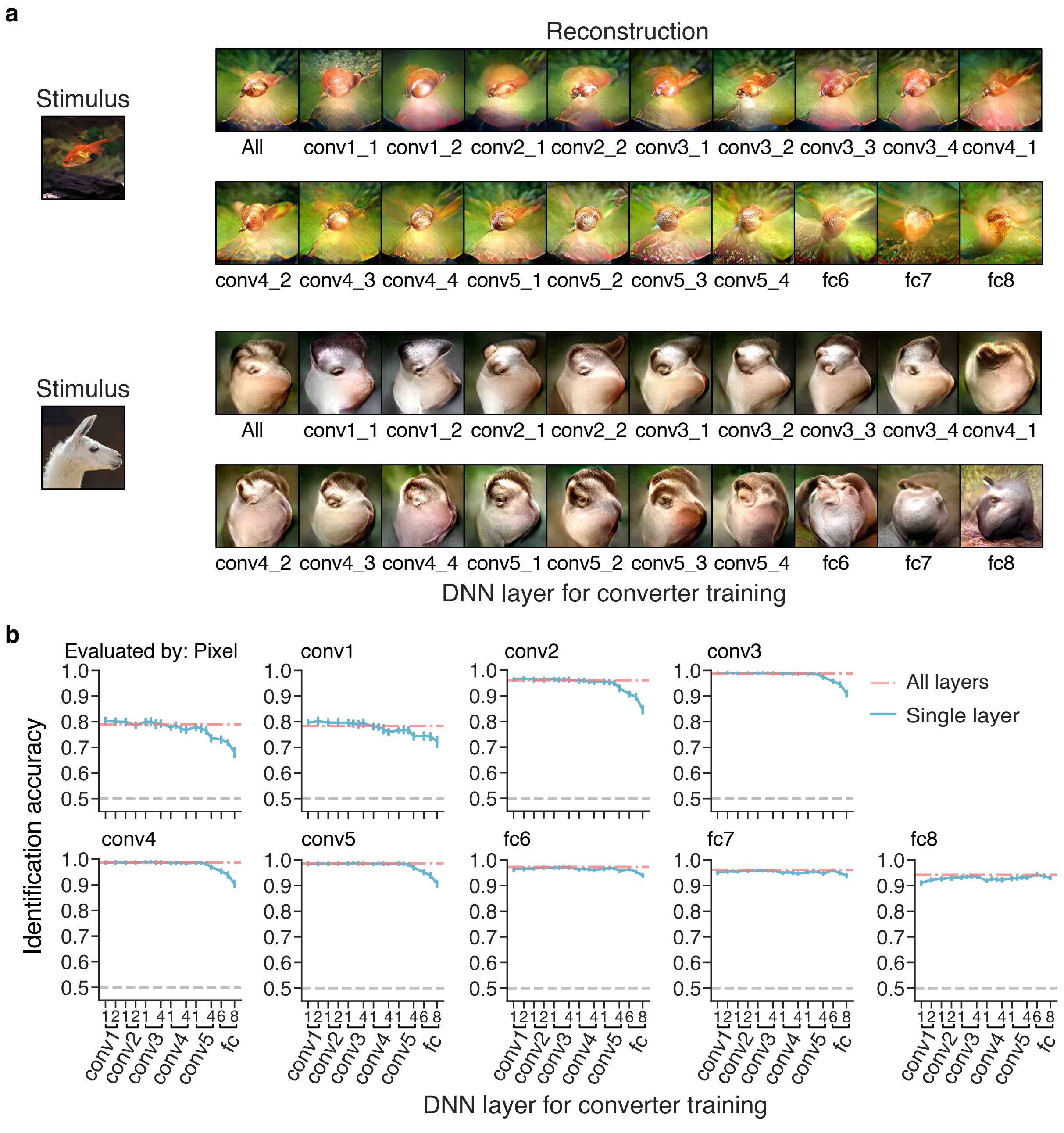

Supplementary Figure 19 | Reconstruction performance with converters trained using the loss from different DNN layers. **a**, Reconstructions. All reconstructed images are generated from the same pair (VC; source: Subject 1, target: Subject 2). For copyright reasons, one stimulus image has been replaced with a visually similar alternative. **b**, Identification accuracy. Pairwise identification was performed using the pixel values and the extracted DNN feature values (AlexNet) from the reconstructions. The "All layers" refers to converters trained using the loss from all DNN layers, while the "single layer" corresponds to converters trained using the loss from single DNN layers. The mean accuracy was calculated in each condition (VC; error bars, 95% C.I. from 20 pairs; dashed lines, 50% chance level).

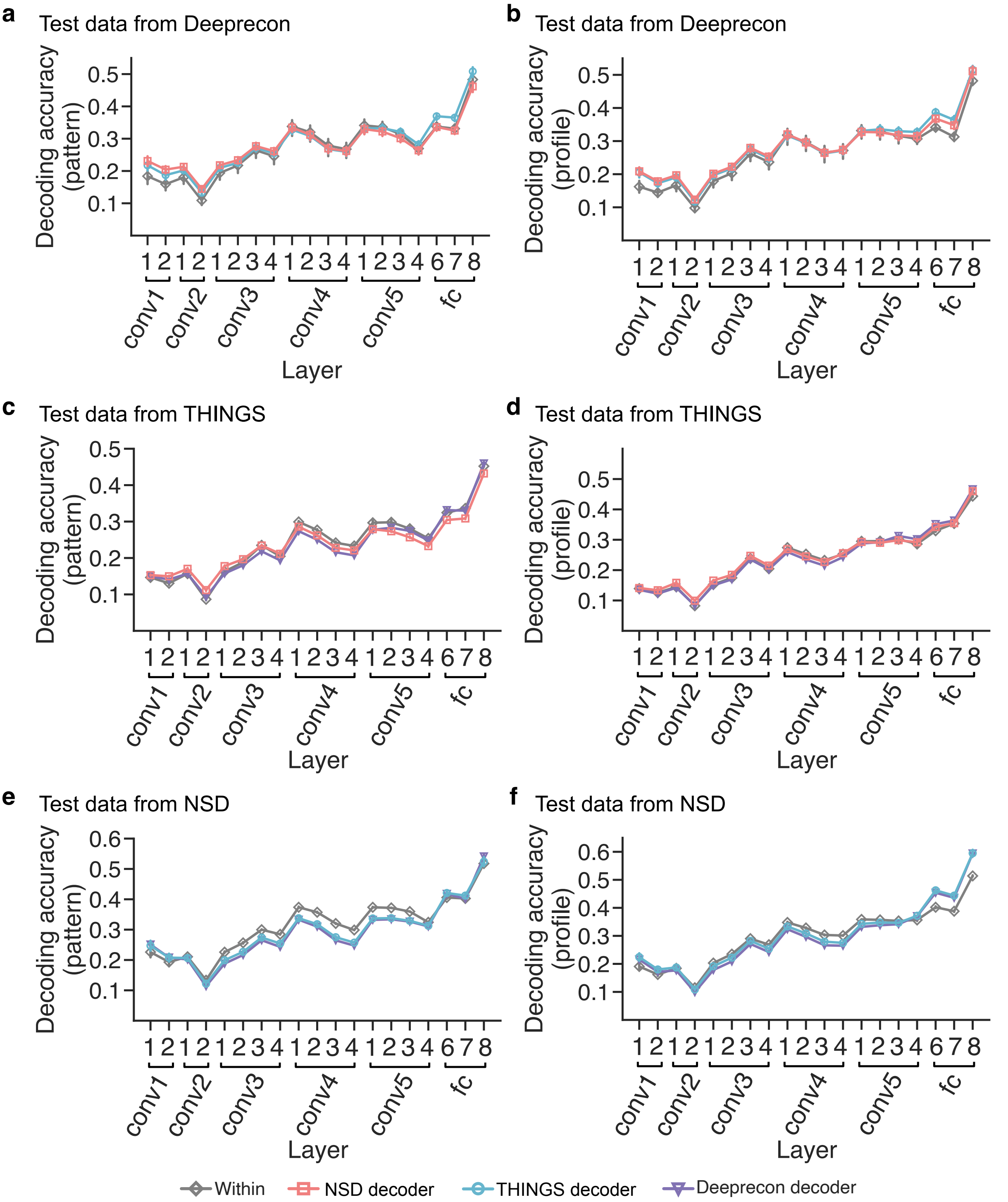

Supplementary Figure 20 | Inter-site DNN feature decoding accuracies. **a**, Feature decoding accuracy (pattern) for Deeprecon test data. The mean pattern correlation for each layer of the VGG19 model is shown for the Within and Inter-site conditions (VC; error bars, 95% C.I. from five subjects for the Within condition, from 20 pairs for the NSD decoder condition and from 15 pairs for the THINGS decoder condition). **b**, Feature decoding accuracy (profile) for Deeprecon test data. The mean profile correlation for each layer of the VGG19 model is shown for the Within and Inter-site conditions (VC; error bars, 95% C.I. from five subjects for the Within condition, from 20 individual pairs for the NSD decoder condition and from 15 pairs for the THINGS decoder condition). **c**, Feature decoding accuracy (pattern) for THINGS test data. **d**, Feature decoding accuracy (profile) for THINGS test data. **e**, Feature decoding accuracy (pattern) for NSD test data. **f**, Feature decoding accuracy (profile) for NSD test data.

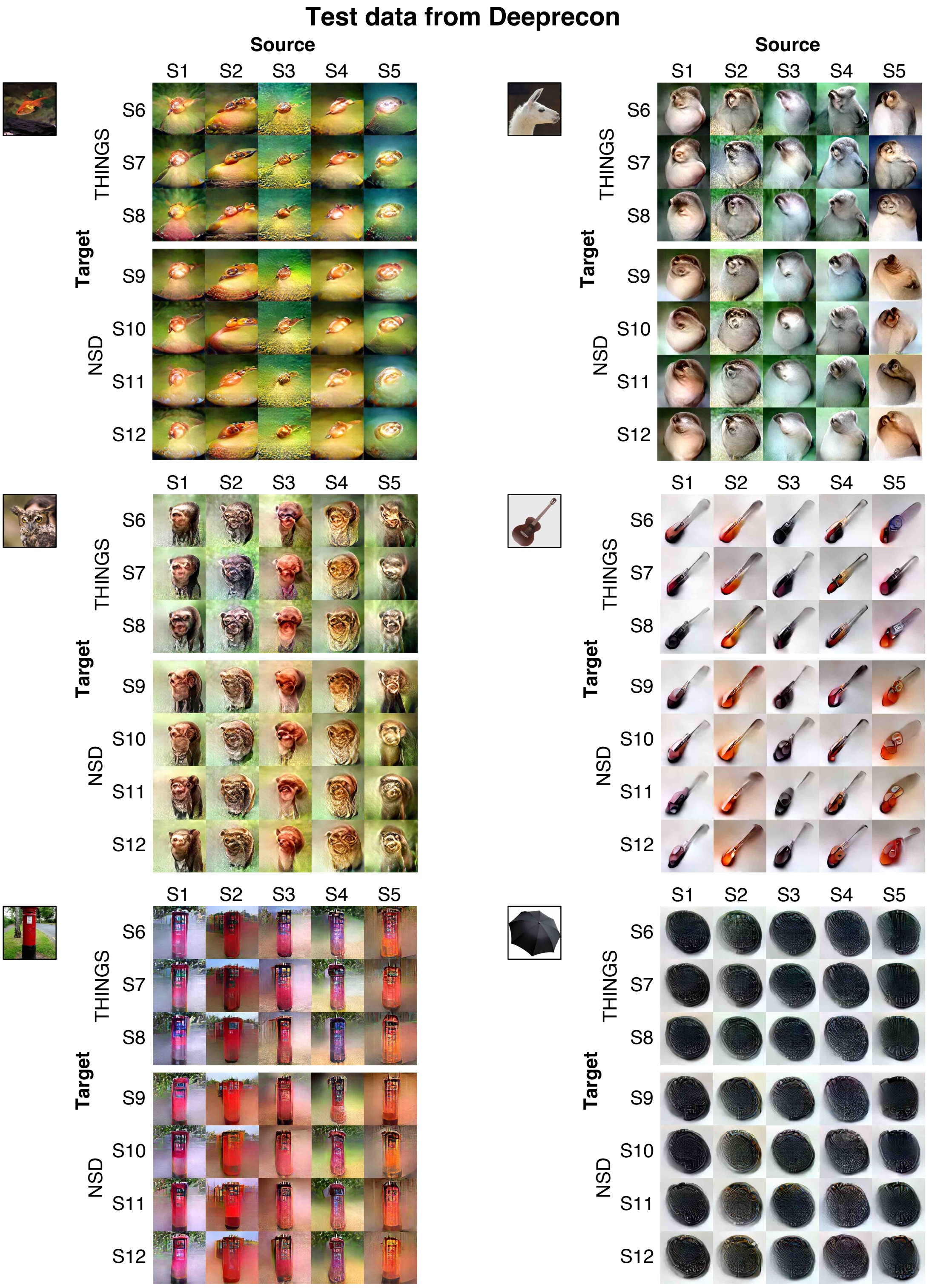

Supplementary Figure 21 | Inter-site image reconstructions for Deeprecon test data. All reconstructions were generated using the whole visual cortex (VC). For copyright reasons, some stimulus images have been replaced with visually similar alternatives.

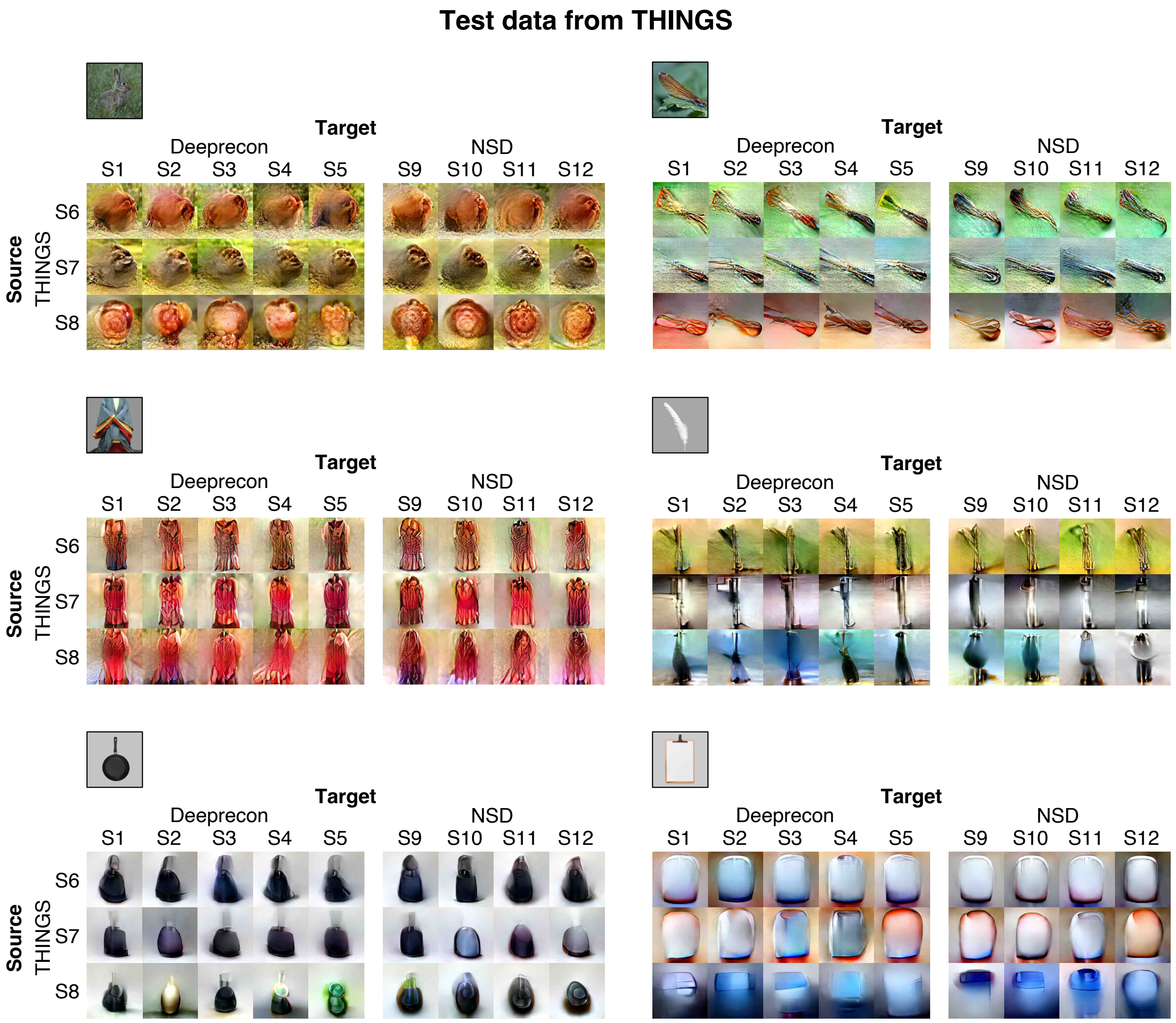

Supplementary Figure 22 | Inter-site image reconstructions for THINGS test data. All reconstructions were generated using the whole visual cortex (VC). For copyright reasons, stimulus images have been replaced with visually similar alternatives.

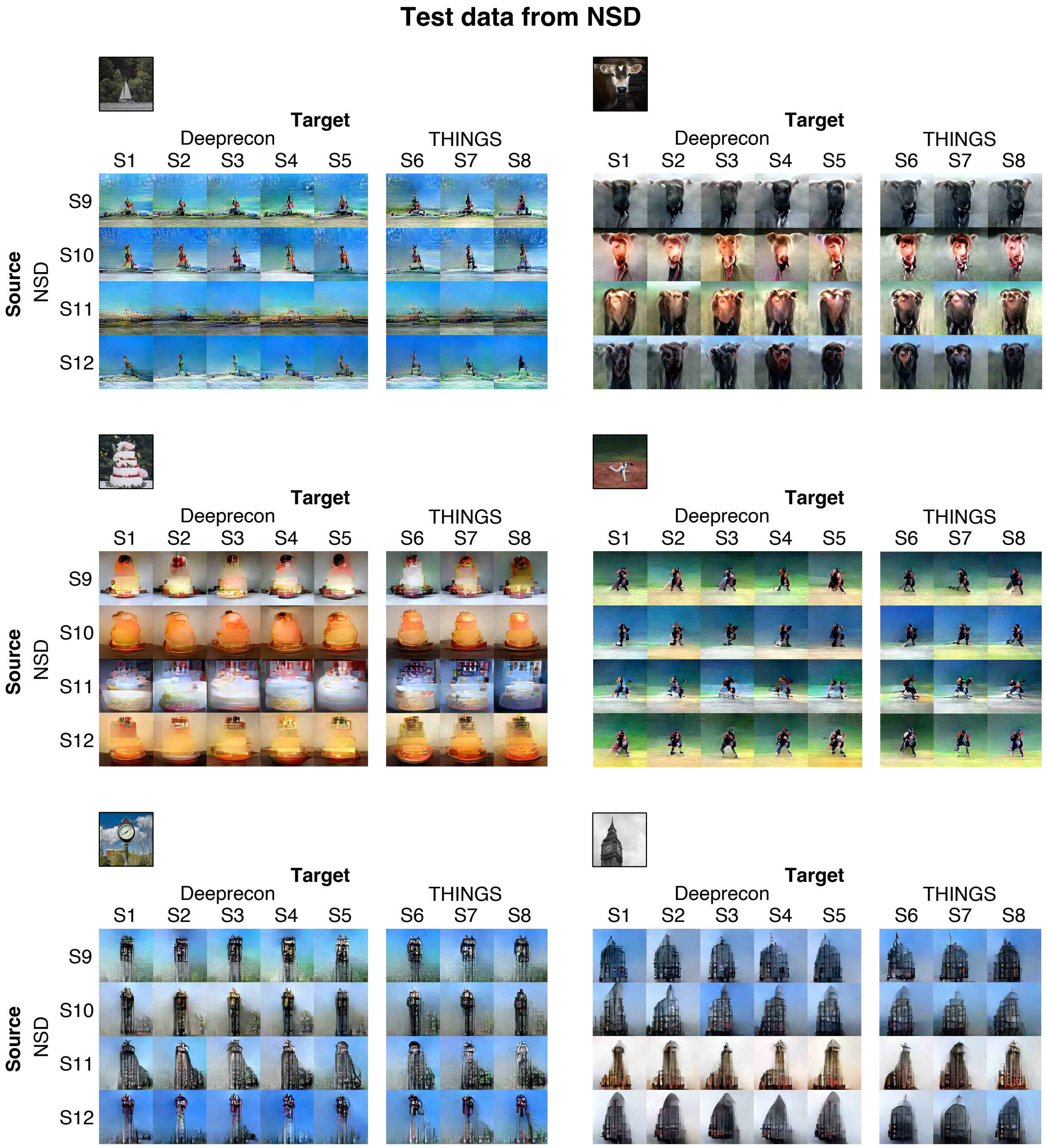

Supplementary Figure 23 | Inter-site image reconstructions for NSD test data. All reconstructions were generated using the whole visual cortex (VC). For copyright reasons, stimulus images have been replaced with visually similar alternatives.

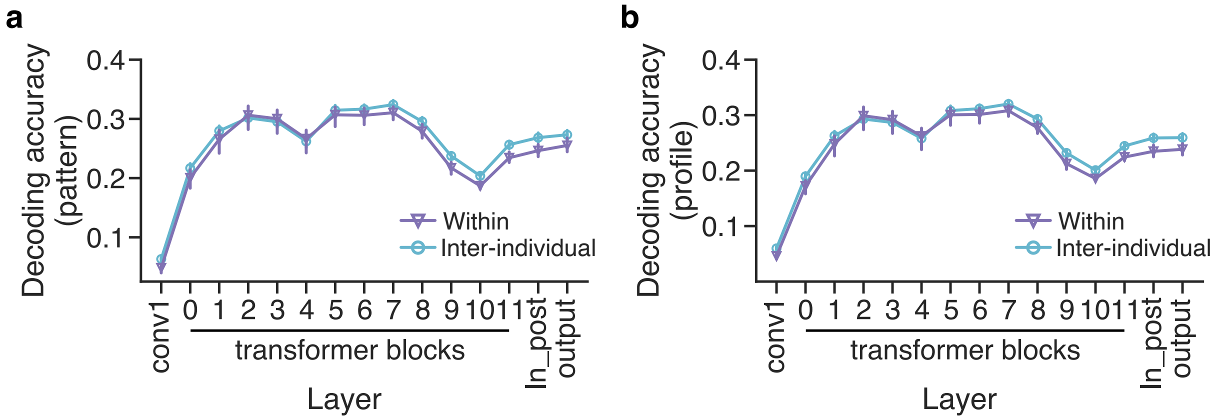

Supplementary Figure 24 | DNN feature decoding performance using the CLIP-ViT decoder. **a**, Pattern correlation. The DNN features from various layers of the CLIP-ViT model are used for analysis, including the conv1 layer, transformer blocks 0-11, the ln_post layer, and the model output layer. The mean pattern correlation for each layer is shown for both the Within condition and the Inter-individual condition (VC; error bars, 95% C.I. from five subjects for the Within condition and from 20 pairs for the Inter-individual condition). **b**, Profile correlation. The two conditions are compared as in **a** but with profile correlation coefficients.

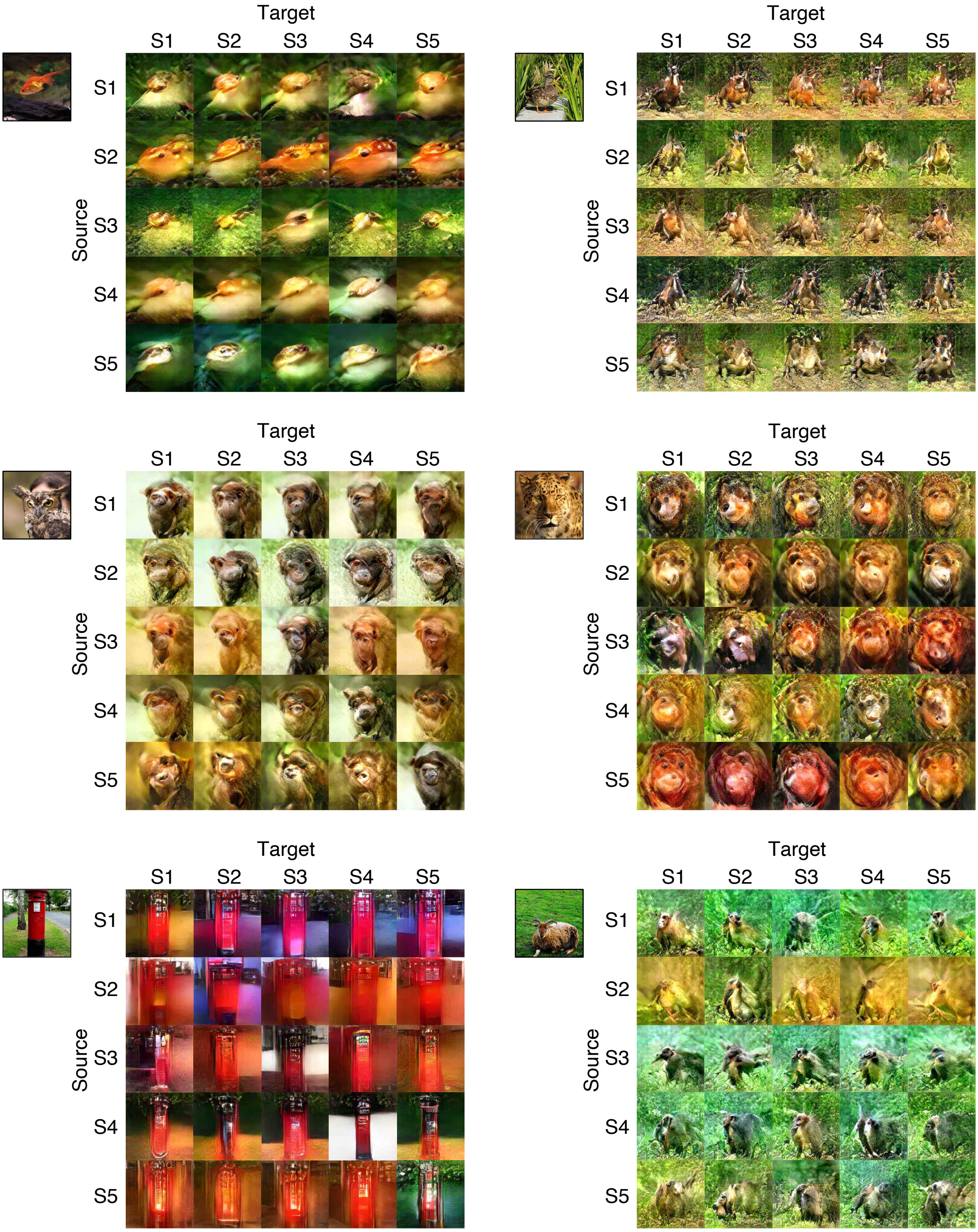

Supplementary Figure 25 | Reconstructed images with the CLIP-ViT decoder for all pairs. The reconstructions for each test stimulus are shown for 20 pairs using the CLIP-ViT decoder. The diagonal images in each block are reconstructed in the Within condition. All reconstructions were generated using the whole visual cortex (VC). For copyright reasons, some stimulus images have been replaced with visually similar alternatives.

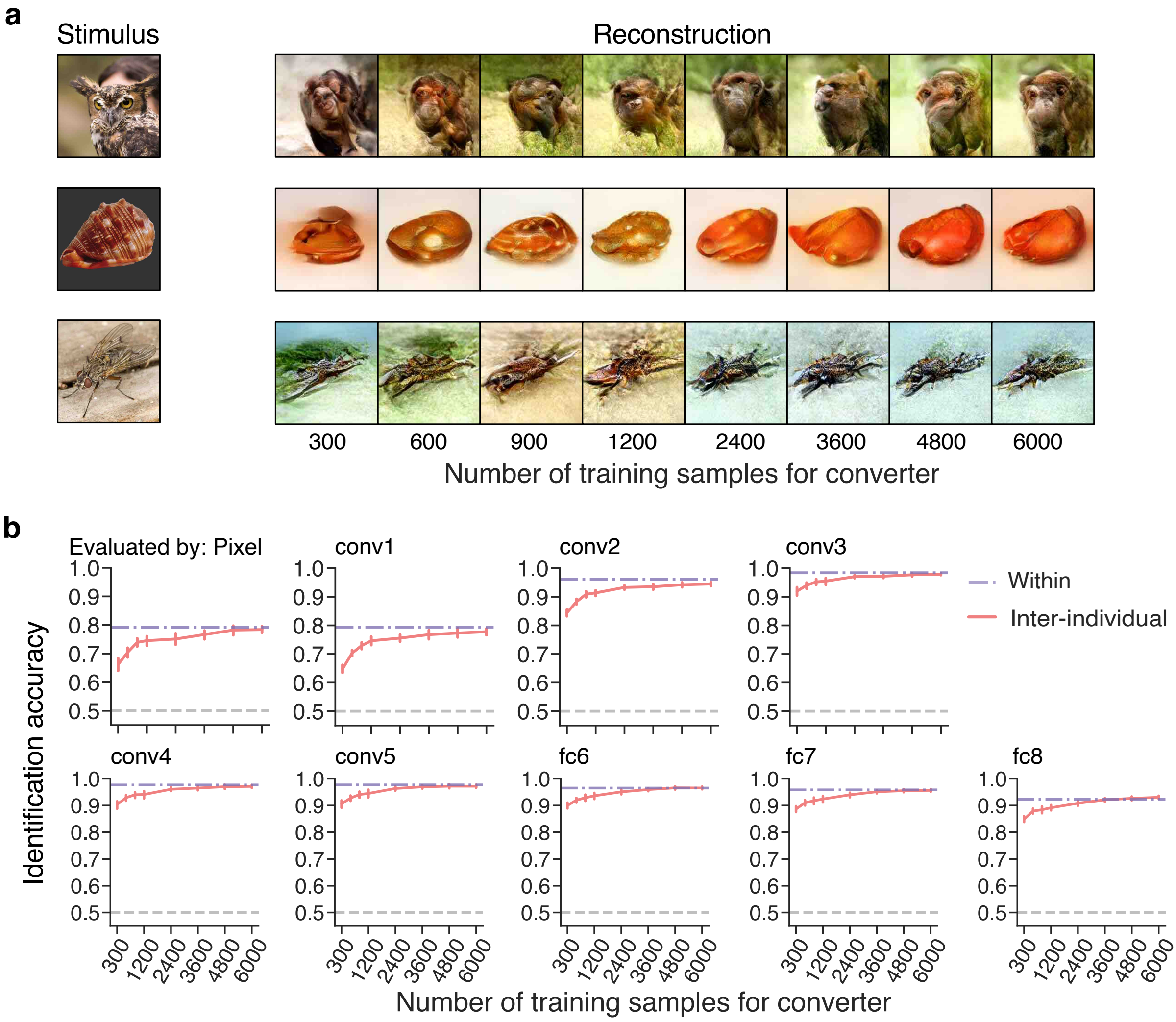

Supplementary Figure 26 | The effect of training sample size for conversion. **a**, Reconstructed images using the CLIP-ViT decoder. The source brain activity was converted into the target's space using VGG19-based converters and was evaluated using the target subject's CLIP-ViT decoder. Reconstructions were generated with a varying number of training data from the same pair (VC; source: Subject 1, target: Subject 2). For copyright reasons, some stimulus images have been replaced with visually similar alternatives. **b**, Identification accuracy. Pairwise identification was performed using the pixel values and the extracted DNN feature values (AlexNet) from the reconstructions with a varying number of training data. The mean identification accuracy was calculated across all individual pairs (VC; error bars, 95% C.I. from 20 pairs; dashed lines, 50% chance level). The results are shown with those from the Within condition.

References

1. Haxby, J. V. *et al.* A common, high-dimensional model of the representational space in human ventral temporal cortex. *Neuron* **72**, 404–416 (2011).
2. Park, J.-Y., Tsukamoto, M., Tanaka, M. & Kamitani, Y. Sound reconstruction from human brain activity via a generative model with brain-like auditory features. *arXiv preprint arXiv:2306.11629* (2023).
3. Iashin, V. & Rahtu, E. Taming visually guided sound generation. *arXiv preprint arXiv:2110.08791* (2021).
