# Supplementary material for: Inter-individual and inter-site neural code conversion without shared stimuli
Source: Nat Comput Sci. 2025 Jul 11;5(7):534–46. doi: 10.1038/s43588-025-00826-5 (PMC12286860; doi:10.1038/s43588-025-00826-5)
Supplement: Supplementary file 2 — Reporting Summary [file 43588_2025_826_MOESM2_ESM.pdf]

nature portfolio

Corresponding author(s): Haibao Wang and Yukiyasu Kamitani

Last updated by author(s): Feb 18, 2025

Reporting Summary

Nature Portfolio wishes to improve the reproducibility of the work that we publish. This form provides structure for consistency and transparency in reporting. For further information on Nature Portfolio policies, see our Editorial Policies and the Editorial Policy Checklist.

Statistics

For all statistical analyses, confirm that the following items are present in the figure legend, table legend, main text, or Methods section.

n/a	Confirmed	
☐	☒	The exact sample size (*n*) for each experimental group/condition, given as a discrete number and unit of measurement
☒	☐	A statement on whether measurements were taken from distinct samples or whether the same sample was measured repeatedly
☐	☒	The statistical test(s) used AND whether they are one- or two-sided<br>*Only common tests should be described solely by name; describe more complex techniques in the Methods section.*
☒	☐	A description of all covariates tested
☒	☐	A description of any assumptions or corrections, such as tests of normality and adjustment for multiple comparisons
☐	☒	A full description of the statistical parameters including central tendency (e.g. means) or other basic estimates (e.g. regression coefficient) AND variation (e.g. standard deviation) or associated estimates of uncertainty (e.g. confidence intervals)
☐	☒	For null hypothesis testing, the test statistic (e.g. *F*, *t*, *r*) with confidence intervals, effect sizes, degrees of freedom and *P* value noted<br>*Give P values as exact values whenever suitable.*
☒	☐	For Bayesian analysis, information on the choice of priors and Markov chain Monte Carlo settings
☒	☐	For hierarchical and complex designs, identification of the appropriate level for tests and full reporting of outcomes
☐	☒	Estimates of effect sizes (e.g. Cohen's *d*, Pearson's *r*), indicating how they were calculated

Our web collection on statistics for biologists contains articles on many of the points above.

Software and code

Policy information about availability of computer code

Data collection	As this study did not involve new data collection, no software was used to collect data.
Data analysis	The data analysis code is written as custom Python scripts (version 3.8.18) and uses the PyTorch library (version 1.7.1) and the Caffe library (version 1.0). The scripts are released publicly on Github (https://github.com/KamitaniLab/InterSiteNeuralCodeConversion).

For manuscripts utilizing custom algorithms or software that are central to the research but not yet described in published literature, software must be made available to editors and reviewers. We strongly encourage code deposition in a community repository (e.g. GitHub). See the Nature Portfolio guidelines for submitting code & software for further information.

Data

Policy information about availability of data

All manuscripts must include a data availability statement. This statement should provide the following information, where applicable:

- Accession codes, unique identifiers, or web links for publicly available datasets
- A description of any restrictions on data availability
- For clinical datasets or third party data, please ensure that the statement adheres to our policy

The data used is publicly available and the sources are as follows:
Deeprecon dataset: https://doi.org/10.18112/openneuro.ds001506.v1.3.1 for subjects 1-3, https://doi.org/10.18112/openneuro.ds003430.v1.2.0 for the dataset of training natural-image session for subjects 4 and 5, and https://doi.org/10.18112/openneuro.ds003993.v1.0.0 for the dataset of test natural-image and artificial-

image sessions for subjects 4 and 5;
THINGS dataset: https://doi.org/10.18112/openneuro.ds004192.v1.0.5;
Natural Scene Dataset:https://naturalscenesdataset.org;
DeepSoundRecon dataset: https://doi.org/10.6084/m9.figshare.23633751.v9.

Human research participants

Policy information about studies involving human research participants and Sex and Gender in Research.

Reporting on sex and gender	The Deeorecon dataset recruited five subjects (four males and one female).<br>The THINGS dataset recruited three subjects (one male and two females).<br>The NSD dataset recruited eight subjects (two males and six females).<br>The DeepSoundRecon dataset recruited five subjects (four males and one female).
Population characteristics	The Deeprecon dataset: age range 25-36 years, subjects with normal or corrected-to-normal vision.<br>The THINGS dataset: mean age: 25.33 years, subjects with normal or corrected-to-normal vision.<br>The NSD dataset: age range, 19–32 years subjects with normal or corrected-to-normal vision.<br>The DeepSoundRecon dataset: mean age: 27.6 years, subjects with normal hearing.
Recruitment	Participants were from publicly available datasets, including Deeprecon, THINGS, NSD, and DeepSoundRecon, all approved by their respective institutional review boards. Informed consent was obtained from all participants prior to data collection. Each dataset has specific inclusion criteria, such as normal or corrected-to-normal vision and no history of neurological or psychiatric disorders (NSD). A potential selection bias may exist, as participants in neuroimaging studies tend to be highly motivated and experienced with experimental tasks, which may impact generalizability.
Ethics oversight	The Deeprecon dataset: approved by the Ethics Committee of the Advanced Telecommunications Research Institute International (ATR).<br>The THINGS dataset: approved by the NIH Institutional Review Board.<br>The NSD dataset: approved by the University of Minnesota Institutional Review Board.<br>The DeepSoundRecon dataset: approved by the Ethics Committee of the Advanced Telecommunications Research Institute International (ATR).<br>Our study is also approved by the Ethics Committee of the Advanced Telecommunications Research Institute International (ATR).

Note that full information on the approval of the study protocol must also be provided in the manuscript.

Field-specific reporting

Please select the one below that is the best fit for your research. If you are not sure, read the appropriate sections before making your selection.

☒ Life sciences ☐ Behavioural & social sciences ☐ Ecological, evolutionary & environmental sciences

For a reference copy of the document with all sections, see nature.com/documents/nr-reporting-summary-flat.pdf

Life sciences study design

All studies must disclose on these points even when the disclosure is negative.

Sample size	The sample size was predetermined by the original dataset providers, as this study used publicly available datasets. These datasets provide 114 subject pairs for our analysis. Power analysis indicates that a minimum of 15 subject pairs is required to detect a large effect size (Cohen's h = 0.8) with 80% power at $\alpha = 0.05$. The current sample size exceeds this requirement, ensuring robust statistical power for our analyses.
Data exclusions	No data were excluded from the analysis.
Replication	The analyses are performed for each subject pair. The effects are replicated across 114 subject pairs.
Randomization	In the analysis of the effect of stimulus overlap between converter and decoder trainings, we randomly divided the training samples from the Deeprecon dataset into two distinct halves based on the categories of stimuli. The source subject were provided with 3000 training samples (600 images from 75 randomly selected categories out of 150 categories, with five repetitions of each image), and the target subject were given a different set of 3000 training samples (the remaining 600 images with five repetitions each). This strategy was designed to prevent overlapping stimuli between the source and target subjects and to avoid any pairing in their brain activity patterns.
Blinding	Blinding was not applicable in this study, as the data were obtained from publicly available datasets, and the investigators were not involved in participant recruitment, data collection, or group allocation.

Reporting for specific materials, systems and methods

We require information from authors about some types of materials, experimental systems and methods used in many studies. Here, indicate whether each material, system or method listed is relevant to your study. If you are not sure if a list item applies to your research, read the appropriate section before selecting a response.

Materials & experimental systems

n/a	Involved in the study
☒	☐ Antibodies
☒	☐ Eukaryotic cell lines
☒	☐ Palaeontology and archaeology
☒	☐ Animals and other organisms
☒	☐ Clinical data
☒	☐ Dual use research of concern

Methods

n/a	Involved in the study
☒	☐ ChIP-seq
☒	☐ Flow cytometry
☐	☒ MRI-based neuroimaging

Magnetic resonance imaging

Experimental design

Design type

The Deeorecon dataset: passiave image-viewing task
The THINGS dataset: passiave image-viewing task
The NSD dataset: passiave image-viewing task
The DeepSoundRecon dataset: passiave sound-listening task

Design specifications

The Deeorecon dataset: Each presentation of an image lasted for 8 s in a stimulus block. fMRI signals were measured while subjects each viewed 1,290 visual images (8,000 trials) over the course of 15–20 scan sessions.
The THINGS dataset: Each image was presented for 0.5 ms, followed by 4 s of eye fixation without image stimuli. fMRI signals were measured while subjects each viewed 8,740 unique visual images (11,040 trials) over the course of 15–16 scan sessions.
The NSD dataset: Images were presented for 3 s with 1-s gaps in between images. fMRI signals were measured while subjects each viewed 9,000–10,000 distinct natural scenes (22,000–30,000 trials) over the course of 30–40 scan sessions.
The DeepSoundRecon dataset: Each sound stimulus was presented for 8 s within a stimulus block. fMRI signals were measured while subjects each listend 1,250 sound stimuli (5,200 trials) over the course of 13–17 scan sessions.

Behavioral performance measures

Not applicable because the passive image-viewing and sound-listening tasks do not involve measurable behavioral performance data.

Acquisition

Imaging type(s)

All imaging types used in this study were functional MRI.

Field strength

The Deeorecon dataset: 3T
The THINGS dataset: 3T
The NSD dataset: 7T
The DeepSoundRecon dataset: 3T

Sequence & imaging parameters

The Deeorecon dataset: An interleaved T2-weighted gradient-echo echo planar imaging (EPI) scan was performed to acquire functional images covering the entire brain (TR, 2000 ms; TE, 43 ms; flip angle, 80 deg; FOV, 192 × 192 mm; voxel size, 2 × 2 × 2 mm; slice gap, 0 mm; number of slices, 76).
The THINGS dataset: The whole-brain functional MRI data was collected with 2 mm isotropic resolution (60 axial slices; 2 mm slice thickness; no slice gap; matrix size 96×96; FOV, 192 × 192 mm; TR, 1.5 s; TE, 33ms; flip angle, 75 deg).
The NSD dataset: The primary fMRI sequence involved gradient-echo EPI, FOV 216 mm x 216 mm, matrix size 120 x 120, slice thickness 1.8 mm, orientation axial, TR 1.6 s, TE 22.0 ms, and flip angle 62 deg.
The DeepSoundRecon dataset: An interleaved T2-weighted gradient-echo echo planar imaging (EPI) scan was performed to acquire functional images covering the entire brain (TR, 2000 ms; TE, 43 ms; flip angle, 80 deg; FOV, 192 × 192 mm; voxel size, 2 × 2 × 2 mm; slice gap, 0 mm; number of slices, 76).

Area of acquisition

Whole-brain scans

Diffusion MRI ☐ Used ☒ Not used

Preprocessing

Preprocessing software

Already preprocessed MRI data were used, based on the preprocessing pipeline of Deeprecon dataset, THINGS dataset, NSD dataset, and DeepSoundRecon dataset. Preprocessing involved tools of fMRIPrep, FreeSurfer6, and selected tools from SPM,

	FSL, ANTs, and MRTrix3.
Normalization	Already normalized MRI data were used, based on the preprocessing pipelines of the DeepRecon dataset, THINGS dataset, NSD dataset, and DeepSoundRecon dataset. These data involved subject-native space and atlas spaces (MNI, fsaverage).
Normalization template	For data in atlas spaces, Normalization template involved the MNI152 and fsaverage templates.
Noise and artifact removal	Already preprocessed MRI data were used, based on the preprocessing pipelines of the DeepRecon dataset, THINGS dataset, NSD dataset, and DeepSoundRecon dataset. For Deeprecon data, the BOLD time series were temporally shifted by 4 s to account for hemodynamic delays and then regressed for nuisance variables. The data samples were finally despiked to reduce extreme values (beyond ±3 SD for each run) in the time series and averaged within each 8-s trial (four volumes). For THINGS data, the ICA denoising was performed,followed by the GLMdenoise method. For the GLM preparation of the NSD data, the data-driven analysis method GLMdenoise and the statistical technique of ridge regression were used. For DeepSoundRecon data, the BOLD time series were temporally shifted by 2 s to account for hemodynamic delays and then regressed for nuisance variables.
Volume censoring	No volume censoring was performed.

Statistical modeling & inference

Model type and settings	Predictive model (i.e. decoding model that predicts representation of the stimulus as a function of brain recordings)
Effect(s) tested	We test whether fMRI activities is predictive of representations of the stimulus

Specify type of analysis: ☐ Whole brain ☒ ROI-based ☐ Both

Anatomical location(s)	The regions of interest (ROIs) were defined based on previous literature (Engel et al., 1994; Sereno et al., 1995; Kourtzi and Kanwisher, 2000; Kanwisher et al., 1997; Epstein and Kanwisher, 1998; Glasser et al., 2016), and the ROI masks were obtained from the dataset.
Statistic type for inference (See Eklund et al. 2016)	Prediction performance was evaluated using Pearson correlation between predicted and actual image features. The mean Pearson correlation and its 95% confidence interval (CI) were computed using bootstrap resampling (1,000 iterations).
Correction	No multiple comparison correction was applied, as statistical inference was based on the 95% confidence interval (CI) obtained via bootstrap resampling.

Models & analysis

n/a	Involved in the study
☒	☐ Functional and/or effective connectivity
☒	☐ Graph analysis
☐	☒ Multivariate modeling or predictive analysis

Multivariate modeling and predictive analysis	Feature extraction: The image stimuli are input into different pre-trained DNN models (including VGG19, AlexNet, and CLIP) to obtain the latent DNN features for decoder training, converter training, and evaluation.<br>Model: The neural code converter for each pair of subjects uses a nonlinear Multi-Layer Perceptron (MLP) to predict the brain activity patterns of one subject (target) from the brain activity patterns of another subject (source).<br>Training: The target subject's training data is used to pre-train the target decoder, whereas the source subject's training data is used for the converter training. The converter is optimized so that the converted brain activity is decoded into content representations that closely resemble that of the stimulus given to the source subject.<br>Evaluation: We evaluted the neural code converter model using three metrics: conversion accuracy, decoding accuracy, and image reconstruction. For conversion accuracy, we computed the Pearson correlation between the predicted brain activities and the measured brain activities for each converter model. For decoding accuracy, we calculated the Pearson correlation between the DNN features decoded from the converted brain activities and the true features from the image stimuli. For image reconstruction, we reconstruced images from the converted brain activities and used identification analysis to measure reconstruction accuracy. This approach involved the identification of the presented image out of two alternatives based on the Pearson correlation of image features, including pixel values and DNN features.
